# Bioprospecting Bioactive Polar Lipids from Olive (*Olea europaea* cv. *Galega vulgar*) Fruit Seeds: LC-HR-MS/MS Fingerprinting and Sub-Geographic Comparison

**DOI:** 10.3390/foods11070951

**Published:** 2022-03-25

**Authors:** Eliana Alves, Felisa Rey, Tânia Melo, Madalena P. Barros, Pedro Domingues, Rosário Domingues

**Affiliations:** 1 Mass Spectrometry Centre, LAQV-REQUIMTE & Department of Chemistry, University of Aveiro, Campus Universitário de Santiago, 3810-193 Aveiro, Portugal; felisa.rey@ua.pt (F.R.); taniamelo@ua.pt (T.M.); p.domingues@ua.pt (P.D.); mrd@ua.pt (R.D.); 2 ECOMARE & CESAM—Centre for Environmental and Marine Studies, Department of Chemistry, University of Aveiro, Campus Universitário de Santiago, 3810-193 Aveiro, Portugal; 3 Cooperativa de Olivicultores de Nelas, C.R.L., Zona Industrial de Nelas, 3520-095 Nelas, Portugal; mmppbarros56@gmail.com

**Keywords:** lipidomics, mass spectrometry, geographical origin, chemical markers, olive seeds

## Abstract

Olive seeds have been considered as a new nutritionally healthy food supplement. They are rich in monounsaturated n-9 and essential polyunsaturated n-6 lipids. However, little is known about their polar lipids, potentially bioactive and chemical identity markers for olive pulp and oil. This work aimed to identify the polar lipidome of olive seeds to find possible bioactive compounds and markers of geographic origin, by studying samples from six Portuguese sub-regions. Polar lipids were obtained by solid/liquid extraction, NH_2_-solid-phase extraction, and identified by hydrophilic interaction liquid chromatography (HILIC)-HR-ESI-MS and MS/MS. Ninety-four compounds were identified, including phospholipids, glycolipids, sphingolipids, and acyl sterol glycosides, several of which bear polyunsaturated fatty acids. Multivariate statistical analysis found unique profiles within each sub-region and markers of geographic identity, primarily phosphatidylcholines, phosphatidylethanolamines, and lysophosphatidylethanolamines. Therefore, polar lipid signatures should be further investigated, to assess their bioactivity, nutritional value, and chemical identity for valuing olive seeds and their oil.

## 1. Introduction

The consumption of fruit seeds and their oils has increased in recent years in Western countries, due to the adoption of healthy eating habits and consumer demand for functional foods. Likewise, agro-industrial by-products, such as fruit seeds, can be highly valued for phytochemicals or oil extraction for human nutrition, or be used as raw materials for the pharmaceutical and cosmetics industries. This is the case with olive seeds.

A new technological approach can separate the pit from the olive pulp during olive processing to further recover the entire seeds [1]. Therefore, olive seeds and their oil are starting to establish themselves (in terms of nutrition and nutraceutical properties) as interesting ingredients and a promising source of phytochemicals. In addition, knowing the lipid composition of olive seeds is essential to identify potential molecular markers of quality, identity, and authenticity in virgin olive oils. Nevertheless, there is a lack of information on the bioactive properties and lipid composition of olive seeds and their oil, namely the plasticity of the lipidome as a function of geographic variation.

Olive seeds are rich in lipids (30–43%, dry weight) [2,3]. They contain 58.4–73.6% of oleic acid (18:1), 17.1–24.2% of linoleic acid (18:2n-6), and 0.1–5.0% of linolenic acid (18:3n-3) of the total fatty acid content [2,3,4,5]. The lipid fraction has ca. 80 to 97% of triacylglycerols [5] and other lipids, such as phytosterols (4.1%), mainly β-sitosterol [6], and 0.1% of phospholipids (PL) [3]. PL are polar lipids, a family that includes many classes and subclasses of molecules, such as glycolipids (GL), sphingolipids (SL), sterol derivatives, among others. Polar lipids are essential constituents of all cell membranes, are involved in vital functions as signaling molecules and cellular mediators and are, nowadays, considered to be high-value lipids due to their emerging health benefits.

Polar lipids, extracted from olive oil and olive pomace, exerted in vitro [7] and in vivo [8,9] antithrombotic and anti-atherosclerotic activities due to inhibitory or antagonistic actions against the platelet aggregation factor (PAF), a potent inflammation mediator. Polar lipids from olives and olive oil have been highlighted for their potential applications in animal feed, food supplements, and food ingredients [10,11]. However, little is known about the polar lipid profile in these matrices [10]. The PL profile of the olive seeds is different from that of olive pulp and depends on the botanical origin [12]. In addition, the geographical, agronomic, and edaphoclimatic characteristics, or the maturation index, influence the lipid profile of the olive fruits and, consequently, of olive oils [13].

The lipidomic study of polar lipids in olive seeds is relevant for the bioprospecting of potential bioactive compounds in these seeds and helps to understand the beneficial effects of olive oil consumption on cardiovascular health, inflammation, and other well-being conditions; to find molecular identity markers that allow one to define a characteristic profile, associated with a variety, a region, or a final product, in terms of authenticity and quality of olives and olive oils; finally, for a better understanding of the lipid fraction in nutritional terms.

Similar studies have used different extraction methods and analytical techniques to identify and characterize the polar lipids of olive fruits. For example, the Folch method has been used for total lipid extraction in Spanish olive fruits [12] and Portuguese olive seeds [14]. Bligh and Dyer’s method was used in Portuguese olive fruit pulp [15]. Both methods were used with some modifications, by varying the volume of solvents, incubation times, and centrifugation parameters. Different approaches have been used for the recovery of polar lipids from total lipid extracts. Some examples include enrichment or fractionation by solid-phase extraction (SPE), using simple silica (Si) columns for olive seeds [14], or weak anionic exchange aminopropyl (NH_2_) columns for olive pulp [15]. For the characterization of polar lipids from the olive pulp in SPE-enriched fractions, in a previous study, we used HILIC-LC-high-resolution-mass spectrometry (HR-ESI-MS) and MS/MS [15]. The polar lipid-rich fraction, obtained with the SPE-Si of Portuguese olive seeds, was analyzed by using thin-layer chromatography (TLC) and direct injection ESI-MS and MS/MS [14]. Montealegre et al. (2013) recovered polar lipids from olive fruits after extracting total lipids with the Folch method and dissolving the lipid extract in methanol with 0.5% (*v/v*) acetic acid, followed by centrifugation and the recovery of the upper phase [12]. The polar lipid fraction was analyzed by non-aqueous capillary electrophoresis coupled with ESI-MS [12].

Following previous work on the polar lipidome of olive pulp [15], this study aimed to identify the polar lipidome of olive seeds of an autochthonous Portuguese cultivar, *Olea europaea* cv. *Galega vulgar*. We used a lipidomic approach based on NH_2_-SPE for total lipid fractionation and HILIC-normal phase-LC-HR-ESI-MS and MS/MS for the analysis of polar lipids. In addition, we studied six olive groves from the same geographic region (Nelas, Portugal) to verify if there is a cultivar-specific polar lipid fingerprint or if the geographic sub-region affects the polar lipid profile of olive seeds.

## 2. Materials and Methods

### 2.1. Chemicals and Lipid Standards

The following solvents were used: chloroform (CHCl_3_), methanol (MeOH), *n*-hexane, acetonitrile and HPLC-grade diethyl ether (Fisher Scientific Ltd., Loughborough, UK). Other reagents were obtained from main commercial sources. The ultra-pure water was type I, with a resistivity of 18.2 MΩ·cm at 25 °C (Synergy, Millipore Corporation, Billerica, MA, USA). PL internal standards were the following: 1,2-dimyristoyl-*sn*-glycero-3-phosphate (dMPA); 1,2-dimyristoyl-*sn*-glycero-3-phosphoethanolamine (dMPE); 1,2-dimyristoyl-*sn*-glycero-3-phosphocholine (dMPC); 1-nonadecanoyl-2-hydroxy-*sn*-glycero-3-phosphocholine (LPC); *N*-palmitoyl-D-erythro-sphingosylphosphorylcholine (NPSM); 1,2-dimyristoyl-*sn*-glycero-3-phospho-1-serine (dMPS); 1,2-dipalmitoyl-*sn*-glycero-3-phosphatidylinositol (dPPI); 1,1′,2,2′-tetramyristoyl cardiolipin; 1,2-dimyristoyl-*sn*-glycero-3-phospho-(1′-*rac*-glycerol) (dMPG); *N*-heptadecanoyl-D-erythro-sphingosine [C17 Cer (d35:1)]. All these standards were acquired from Avanti Polar Lipids, Inc. (Alabaster, AL, USA).

### 2.2. Samples

Olive samples (cv. *Galega vulgar*) were collected at random in December 2016, during the 2016/2017 campaign, in six traditional rainfed olive groves, from five trees in each grove, in the Nelas region (Nelas, Portugal, 40.749711, −7.853651) (Supplementary Material, Figure S1 and Table S1). Nelas is a Portuguese village in the district of Viseu. Three olive groves were selected in Vilar Seco, the location with the highest density of olive groves. Three other olive groves were chosen in locations equidistant from Vilar Seco and equidistant from each other, at Silgueiros (Sil), Oliveira de Barreiros (OB), and Vila Ruiva (VR) and Table S1).

This study was designed to characterize the polar lipidome of samples from three olive groves from the same sub-region (Vilar Seco: VS_1, VS_2, VS_3) and three olive groves from three other sub-regions (Sil, OB, VR) and check for significant differences between the six olive groves. In Vilar Seco, it is assumed that the most significant number of edaphoclimatic variables are controlled, being as similar as possible (soil composition, temperature, altitude, precipitation, among others). The dependent variable is the geographic location of the cv. *Galega vulgar* olive trees. If all independent variables are held constant, the polar lipidome of the olive seeds should not show any significant difference between the olive groves. Testing different olive groves in the same location (VS) and three other olive groves in other nearby locations (Sil, OB, and VR) will allow assigning a polar lipid fingerprint to olive seeds cv. *Galega vulgar* or finding different fingerprints depending on the geographic origin.

The olive samples were transported to the laboratory at 4 °C in cooling boxes after being hand-picked. Damaged, sick, or spotted fruits were discarded. Then, the selected fruits were washed with tap water, distilled water, and dried with a cloth. The pulp was removed from the fruit with a knife, and the stones were softly crushed with a hammer to collect the seeds. Finally, the seeds were stored until total lipid extraction in a glass vial at −20 °C.

### 2.3. Total Lipid Extraction

Total lipids from the olive seeds were extracted through the Folch method [16]. First, the seeds (ca. 300 mg) were macerated with 6.5 mL of CHCl_3_/MeOH (2:1, by volume) using an ice-cold mortar and pestle. The mixture was well-homogenized, transferred to a chilled glass tube, and stirred on ice for three hours. Then, 1 mL of ultra-pure water was added to induce a two-phase system. The mixture was centrifuged for 10 min at 142× *g* (Mixtasel, JP Selecta, S.A., Barcelona, Spain), and the lipid-containing organic phase was transferred to another glass tube. Lipid extracts were dried under N_2_ and stored in amber glass vials at −20 °C until quantification and fractionation. This procedure was performed in five independent samples (*n* = 5) on the same day for each of the six groups. The extraction yield was determined by gravimetry.

### 2.4. Quantification of Phospholipids and Glycolipids

PL were quantified using the phosphorus assay [17]. This procedure was performed for each total lipid extract and polar lipid-rich fractions obtained after fractionation by SPE. Initially, 20 μg of total lipid extract dissolved in CHCl_3_ was transferred to glass tubes in duplicate, and the CHCl_3_ was evaporated under an N_2_ stream. Then 125 µL of 70% perchloric acid was added and placed at 180 °C in a heating block (SBH200D/3, Stuart, Bibby Scientific Ltd., Stone, UK) for 40 min. In parallel, a calibration curve (0, 0.1, 0.2, 0.4, 0.7, 1.0, 1.5, and 2.0 μg·mL^−1^) of an aqueous solution of NaH_2_PO_4_ (100 mg·mL^−1^) was prepared. After the acidic digestion of the samples, 825 μL of ultra-pure water, 125 μL of 2.5% (NH_4_)_6_Mo_7_O_2_4·4H_2_O aqueous solution, and 125 μL of 10% aqueous ascorbic acid were added. The same was done for the phosphorus standards, varying the volumes of ultra-pure water. Next, all tubes were placed in a boiling water bath (Precisterm, JP Selecta, Barcelona, Spain) for 10 min and then allowed to cool. Absorbances were read at 797 nm in a UV-visible spectrophotometer (Multiskan GO, Thermo Scientific, Hudson, NH, USA). The calibration curve was drawn with the absorbance values of the standards to determine the phosphorus amount. The value obtained (in μg of inorganic phosphorus) was multiplied by 25 to estimate the PL amount, a conversion factor representing the average mass of a PL when divided by the phosphorus mass (m = 30.97 g·mol^−1^).

GL were quantified in the total extracts by the orcinol method [18]. First, 50 μg of total lipid extract dissolved in CHCl_3_ was transferred to glass tubes, and the CHCl_3_ was evaporated under N_2_. In parallel, a calibration curve of D-glucose was prepared (0, 2.0, 4.0, 8.0, 16, 20, 30 and 40 mg·μL^−1^) in absolute ethanol (2.0 mg·mL^−1^). Then, 1.0 mL of orcinol solution (1,3-dihydroxy-5-methylbenzene, 0.2% in 70% H_2_SO_4_) was added to each tube. Tubes were heated to 80 °C for 20 min and then cooled to room temperature. Absorbance was read at 505 nm. Next, a calibration curve was drawn with the absorbance values of the standards to determine the amount of glucose. Finally, the obtained value (in μg of glucose) was multiplied by the conversion factor 100/35 to estimate the GL amount [19,20].

### 2.5. Solid-Phase Extraction of the Total Lipid Extracts

To fractionate the total lipid extracts of the olive seeds we used the same procedure as previously used for the olive pulp [15] which had been adapted from Ruiz et al., 2004 [21]. The lipid extracts were fractionated into neutral lipids, intermediate polarity lipids, and polar lipids using NH_2_-SPE cartridges (Discovery DSC-NH_2_, ref. 52637-U Supelco, Sigma-Aldrich, Darmstadt, Germany). The columns were coupled to a vacuum manifold (Visiprep SPE Vacuum Manifold, ref. Supelco-57030-U, Sigma-Aldrich). In brief, the SPE-NH_2_ cartridge was conditioned with *n*-hexane (7.5 mL) and loaded with the lipid extract (15 mg dissolved in CHCl_3_). The lipid elution was as follows: neutral lipids (fraction 1) were eluted with 20 mL CHCl_3_; intermediate polarity lipids were eluted with 12 mL diethyl ether/acetic acid (98:2, by volume, fraction 2); polar lipids were eluted with 6 mL CHCl_3_/MeOH (1:6, by volume, fraction 3) and 3 mL CHCl_3_/MeOH (1:1, by volume, fraction 4). The recovered fractions were collected, evaporated to dryness with N_2_, and transferred to glass vials. PL were quantified in the polar-lipid-rich fractions (combined fractions 3 and 4) before HPLC-MS analysis.

### 2.6. Polar Lipid Analysis by HPLC-MS and HPLC-MS/MS

The polar-lipid-rich fraction was analyzed by HPLC-ESI-MS and HPLC-ESI-MS/MS with a HILIC column. The HPLC system (UltiMate 3000 UHPLC, Thermo Fisher Scientific, Germering, Germany) was coupled online to a Q-Exactive HF hybrid quadrupole-Orbitrap mass spectrometer (Thermo Fisher, Scientific, Bremen, Germany). The mobile phase used a gradient profile and consisted of two eluents: acetonitrile/MeOH/water, 50:25:25, by volume, with 2.5 mM ammonium acetate (A), and acetonitrile/MeOH, 60:40, by volume, with 2.5 mM ammonium acetate (B). Initially, 10% of A was held isocratically for 2 min., linearly increased to 90% of A within 13 min., and a maintenance period of 2 min., returning to the initial conditions in 10 min. Five μL of each sample was added into the HPLC column (Ascentis Si HPLC Pore column, 10 cm × 1 mm, 3 μm, Sigma-Aldrich), with a flow rate of 50 μL min^−1^, at 35 °C. The sample comprised 5 μg of polar lipids dissolved in CHCl_3_, 4 μL of a mixture of PL standards [dMPC, 0.02 μg; dMPE, 0.02 μg; NPSM, 0.02 μg; LPC, 0.02 μg; dPPI, 0.08 μg; dMPG, 0.012 μg; dMPS, 0.04 μg; dMPA, 0.08 μg; C17 Cer(d35:1), 0.04 μg], and 91 μL of eluent B. For the full scan MS run, five injections were performed, corresponding to each biological replicate from each group (*n* = 5). Pools of the five replicates from each group were used for the MS/MS. The spectrometer operated simultaneously in the positive (electrospray voltage was 3.0 kV) and negative (electrospray voltage was −2.7 kV) ionization modes. The resolution was 70,000 and the automatic gain control (AGC) target was 1 × 10^6^. The capillary temperature was 250 °C, and the sheath gas flow was 15 arbitrary units. A resolution of 17,500 and an AGC target of 1 × 10^5^ were used in the MS/MS experiments. The cycles consisted of one full scan mass spectrum and ten data-dependent MS/MS scans continuously repeated throughout the experiments. Dynamic exclusion was 60 s and intensity threshold was 2 × 10^4^. The normalized collision energy applied ranged between 20, 25, and 30 eV.

### 2.7. Data Analysis

Data acquisition was carried out using the Xcalibur data system from Thermo Fisher Scientific (V3.3, Waltham, MA, USA). Polar lipid molecular species were identified both by assignment of the precursor ions observed in LC-MS spectra and by the identification of the well-described fragmentation pattern of each class observed in the MS/MS spectra of each ion, as described in the literature, expected retention time, and mass accuracy with an error of ≤5 ppm. The raw data processing was achieved by using the MZmine software V2.32 [22,23]. Initially, the mass list was filtered, then peaks were detected and processed. The parameters set for MZmine 2.32 were the following: minimum peak height, above 1 × 10^4^; mass accuracy, 5 ppm; join alignment; allowable error of retention time, 0.5 min; acceptable error of *m/z* (*m/z* tolerance), 5 ppm. Peak assignment and ion identification were based on mass accuracy and performed against in-house polar lipid databases based on the database from LIPID MAPS. An online exact mass calculator (https://www.sisweb.com/referenc/tools/exactmass.htm accessed date 9 July 2021) was used for calculating the exact mass values, while the mass errors (≤5 ppm) were determined using a free web calculator (https://warwick.ac.uk/fac/sci/chemistry/research/barrow/barrowgroup/calculators/mass_errors/ accessed date 9 July 2021). The identification of the lipid species was based on the exact mass measurements (error ≤ 5 ppm), the retention time, and the analysis of MS/MS spectra of each ion. Illustrative MS/MS spectra of all classes can be found in Supplementary Material, Figures S2–S7. Next, the relative quantification of the polar lipid species was performed using each ion’s chromatographic peak area values. Finally, data were normalized by dividing the areas of the ions corresponding to the lipid species of each class by the area of the ions assigned as the internal standard of each class [23]. As for hexosylceramide (HexCer), monogalactosyldiacylglycerol (MGDG), digalactosyldiacylglycerol (DGDG), and acyl sterol glycoside (ASG) classes no internal standard was used, the molecular species were normalized using the ceramide internal standard [Cer (d18:1/17:0)] that had the same retention time. Likewise, the lysophosphatidylethanolamine (LPE) class was normalized while using the phosphatidylethanolamine (PE) standard [PE (14:0/14:0)]. The nomenclature and shorthand notation follows the recent LIPID MAPS consensus on classification for MS-derived lipid structures [24].

### 2.8. Statistical Analysis

A one-way analysis of variance (ANOVA) with Tukey’s HSD multiple comparisons (post hoc) test was performed to determine potentially significant statistical differences on the total lipids, total PL, total GL, and PL plus GL contents of olive seeds collected from different groves. Differences in the relative abundance of polyunsaturated, monounsaturated, and saturated lipid species of the four polar lipid families (PL, GL, SL, and ASG) and the relative abundance of the oxygenated PC (oxPC), oxygenated LPC (oxLPC), Cer and HexCer of olive seeds from different locations were also tested using one-way ANOVA and Tukey’s HSD post hoc comparisons. Shapiro–Wilk test and Levene’s test checked for normality and homogeneity of variances, respectively. These statistical analyses were performed using R version 4.0.1 [25] in RStudio version 1.1.442 (RStudio Team, 2016). The statistical significance level was *p* < 0.05.

An analysis of similarities (ANOSIM) analysis with the lipidomic data from the six olive groves was performed to identify differences between sampling locations: VS_1, VS_2, VS_3, Sil, OB, and VR, using sampling location as a factor. Before statistical analysis, the raw data matrix of lipid species normalized areas was log(x + 1) transformed to down-weight the contributions of quantitatively dominant molecular species. Following this transformation, a new matrix was assembled using Euclidean distance. Primer v6.1 was used for performing these analyses [26].

Lipidomic data assessment was performed using Metaboanalyst 5.0 [27]. Missing values were replaced by 1/5 of the minimum positive values of their corresponding variables. A data filtering process was performed to remove low-repeatability variables, using the relative standard deviation (RSD = SD/mean). To down-weight high-abundance molecular species, data were logarithmically transformed and autoscaled before analysis. A heatmap was performed using Euclidean distance measure and Ward clustering algorithm to identify the molecular species that contributed the most to the differences between groups. Principal component analysis (PCA) was performed to visualize the general 2D clustering of samples from the six different olive groves cv. *Galega vulgar*: VS_1, VS_2, VS_3, Sil, OB, and VR. A one-way ANOVA test followed by Tukey’s HSD *post hoc* comparisons was performed to compare the normalized extracted ion chromatograms (XIC) areas of lipid species from different locations. Next, *p*-values were corrected for multiple testing using Benjamini–Hochberg false discovery rate (FDR, *q* values). The 25 polar lipids with the lowest *q* values (i.e., the polar lipid species with the higher discriminating power) were used to calculate the hierarchical clustering heatmap and PCA. All data represent mean ± standard deviation (*n* = 5).

## 3. Results

Untargeted LC-MS/MS determined the detailed fingerprinting of the polar lipids from olive seeds. Univariate and multivariate statistical analyses were performed to verify if the geographic location influences the composition. Samples from six traditional olive groves of the *Galega vulgar* variety, from a Portuguese region, were used (Supplementary Material, Figure S1).

### 3.1. Total Contents of Lipids, Phospholipids, and Glycolipids

**Figure 1:**
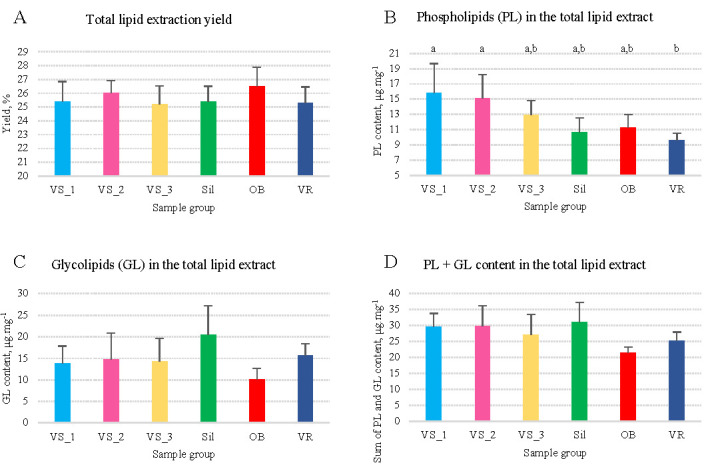
Content of total lipids (**A**), phospholipids (**B**), glycolipids (**C**), and phospholipids plus glycolipids (**D**) in olive seeds from different sub-regions of Nelas, Portugal. Different superscript letters in (**B**) indicate significant differences between groups (one-way ANOVA, Tukey’s HSD post hoc analysis, *p* < 0.05). Samples were collected from six olive orchards located in Vilar Seco_1 (VS_1), Vilar Seco_2 (VS_2), Vilar Seco_3 (VS_3), Silgueiros (Sil), Oliveira de Barreiros (OB), and Vila Ruiva (VR).

The yield of olive seed oil obtained from olive seeds after lipid extraction accounted for 25.6 mg of lipids per 100 g of biomass, varying from 25.2 to 26.5% (Figure 1A and Supplementary Material, Table S2). The PL content in the olive seed oil varied between 9.56 and 15.78 μg·mg^−1^ oil (Figure 1B), with an average of 12.5 μg·mg^−1^ of oil (Supplementary Material, Table S2). The lowest values were observed for the VR group and the highest for the VS_1 and VS_2 groups (Figure 1B). The GL content of olive seed oil varied between 10.14 and 20.52 μg·mg^−1^ of oil (Figure 1C), with an average of 14.9 μg·mg^−1^ of oil (Supplementary Material, Table S2). The lowest and highest values were observed for the OB and Sil groups, respectively (Figure 1C). The sum of PL plus GL contents was 27.4 μg·mg^−1^ oil, on average (Figure 1D). The PL/GL ratio ranged between 0.59 and 1.25, with an average of 1.0, evidencing a higher PL content than GL in the VS and OB groups (Supplementary Material, Table S2). No significant differences were found between the six groups in the total lipid content, total GL content, the sum of PL and GL, or the PL/GL ratio (*p* > 0.05). However, the total PL content was significantly different between VS_1 vs. VR (ANOVA, *p* = 0.015) and VS_2 vs. VR groups (ANOVA, *p* = 0.039).

### 3.2. Polar Lipid Fingerprint

In total, 10 polar lipid classes and 94 lipid species were identified: 56 PL, 17 GL, 16 SL, and 5 ASG (Supplementary Material, Table S3). The number of PL species ranged between 49 and 56 species within the different sample groups, followed by the GL, ranging from 16 to 17 lipid species, the SL between 13 and 15 lipid species, and sterol lipids with 4 to 5 lipid species (Supplementary Material, Table S4).

Five PL classes were identified in all sample groups: phosphatidylcholine (PC), lysoPC (LPC), PE, LPE, and phosphatidylglycerol (PG). The highest number of lipid species was identified in the PC class (22 to 28 species), followed by the PE class (10 to 12 species). The neutral GL classes, identified in the olive seeds, were MGDG and DGDG (eight to nine species). Cer and HexCer were both identified in the olive seeds (up to nine species). Acyl sterol glycosides (ASG) were also found in the polar lipid-rich fraction of the olive seeds (four to five species) (Supplementary Material, Table S4).

Most of the PL molecular species contained the 18:1 acyl chain, but also 16:0 and the essential PUFA 18:2 and 18:3 (Supplementary Material, Table S3). MGDG and DGDG were mainly esterified to 18:3 and 18:2 essential PUFA, but also 18:1, 16:0, and 18:0 fatty acids. In Cer and HexCer, both di- and tri-hydroxy long-chain bases were present as d18:1 or 8-sphingenine, d18:2 or 4,8-sphingadienine, t18:0 or 4R-hydroxysphinganine, and t18:1 or 4-hydroxy-8-sphingenine. ASG were all sitosterol derivatives, and the fatty acyl chains had C16 and C18 up to three double bonds (Supplementary Material, Table S3).

**Figure 2:**
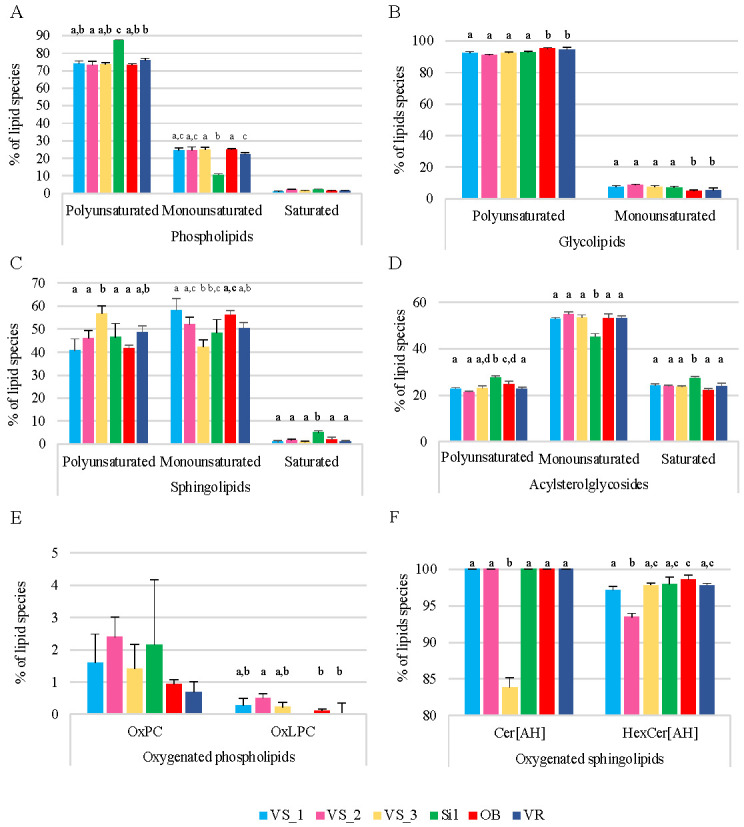
Percentage of polyunsaturated (Poli), monounsaturated (Mono), and saturated (Sat) lipid species by polar lipid category: phospholipids (**A**), glycolipids (**B**), sphingolipids (**C**), and acyl sterol glycosides (**D**), and percentage of oxygenated lipid species in PC and LPC classes (oxPC and oxLPC, respectively) (**E**) and ceramides and hexosylceramides bearing 2-hydroxy fatty acids (Cer[AH] and HexCer[AH], respectively) (**F**). The lipid species were identified in the olive seeds in the six sub-regions of Nelas, Portugal, calculated based on the relative abundance of each lipid species, i.e., normalized extracted-ion chromatogram areas. Different letters on the top of the bars represent significant differences between groups, while the same letter means there is no significant different between the respective groups (one-way ANOVA, Tukey’s HSD post hoc analysis, *p* < 0.05). Samples were collected from six olive orchards located in Vilar Seco_1 (VS_1), Vilar Seco_2 (VS_2), Vilar Seco_3 (VS_3), Silgueiros (Sil), Oliveira de Barreiros (OB), and Vila Ruiva (VR).

The percentages of monounsaturated, polyunsaturated, and saturated lipid species in each category of polar lipids (PL, GL, SL, and ASG) were also assessed by considering the normalized areas of the XIC of each ion and summing the areas by groups within the respective category (Figure 2). Within the different polar lipid categories, PL had a representative fraction of polyunsaturated species (70 to 85%), 10 to 25% of monounsaturated species, and ca. 5% of saturated species (Figure 2A). The GL were mostly polyunsaturated, with about 90% of the lipid species having a sum of at least two double bonds, and ca. 10% were monounsaturated (Figure 2B). On the other hand, there was a predominance of monounsaturated species in the SL (40 to 60%), 40 to 60% polyunsaturated species, and about 5% saturated species (Figure 2C). ASG had 20 to 30% saturated species, 20 to 30% polyunsaturated species, and 45 to 55% monounsaturated species (Figure 2D).

There were significant differences in the sum of the abundances of the four categories of polar lipids (PL, GL, SL, and ASG), when comparing the six groups of samples by lipid category (ANOVA, *p* < 0.05, Figure 2). In the PL family, differences were observed in the abundance of polyunsaturated and monounsaturated lipid species in the Sil group compared to the other sample groups, being more abundant and less abundant than the others, respectively. In the GL family, the OB and VR groups showed the greatest abundance of polyunsaturated lipid species compared to the other groups, the reverse occurring in monounsaturated lipid species (ANOVA, *p* < 0.05, Figure 2). The VS_3 group stood out in the SL family, with the polyunsaturated species showing a higher abundance and the monounsaturated species showing a lower abundance than the other groups (ANOVA, *p* < 0.05, Figure 2). The Sil group was also remarkable in the SL group, having saturated species in greater abundance than the other groups (ANOVA, *p* < 0.05, Figure 2). Finally, in the ASG family, the polyunsaturated and the saturated lipid species were significantly more abundant in the Sil group, and the monounsaturated lipid species were significantly lower than in the other groups of samples (ANOVA, *p* < 0.05, Figure 2). In general, there was significant variability in the degree of saturation of the lipid species, within each family and between groups of samples, indicating heterogeneity in lipid composition among groups.

Oxygenated lipids were found in the PL and SL classes. Some oxPC molecular species had long-chain hydroxy and dihydroxy fatty acyl chains (Supplementary Material, Table S4). An oxLPC with two additional oxygen atoms was also found (LPC 18:1;O2). Oxygenated PC (oxPC) represented ca. 2.5% of the total PC species in all sample groups, and oxLPC represented less than 0.5% of the total LPC species in all groups, except in the Sil group, where they were not detected (Figure 2E). In Cer and HexCer, the acyl chains ranged from C16 to C26 and were composed of 2-hydroxy fatty acids, except in Cer 34:3;O2 and HexCer 34:2;O2 (Supplementary Material, Table S3). Thus, all the Cer species carried 2-hydroxy fatty acids (Cer[AH]), except on the VS_3 group, and more than 90% of the HexCer were HexCer[AH] (Figure 2F).

### 3.3. Sub-Geographic Comparison

After having characterized the polar lipidome, the aim was to verify whether the geographic variation of the samples influenced the polar lipid composition of the olive seeds. The same lipid classes were identified in the six groups of samples from different sub-regions (Supplementary Material, Table S4). However, each group had a unique polar lipid profile. Table 1 shows the missing (undetected) lipid species in the different groups that made each profile unique, with respect to the presence and/or absence of lipid species. There were missing species in all lipid classes, except MGDG. In the Sil group, 11 of the 22 lipid species common to the other groups were not identified (Table 1).

Multivariate analysis was performed for the lipid species dataset obtained after relative quantification, considering the six olive groves from the four different sub-regions (VS, Sil, OB, and VR) (Supplementary Material, Figure S8). The Sil group was separated from the other groups in the PCA scores, plot along with PC 2. The eigenvalues of the first two principal components represented 41.4% of the total variance, (PC 1 24.1% and PC 2 17.3%. PC 1 allowed us to separate the OB group (negative values of PC 1) from Sil, VS_1, and VS_2 (positive values of PC 1). The VS_3 samples were well grouped in this PCA and distributed along with the positive and negative values of PC 1. The VR samples were more dispersed than in the other groups, slightly overlapping the VS and OB groups. The three VS groups were different but close to each other compared to the other groups. These results indicate that the polar lipid profile of the Sil group differs from the other groups. Although having some similarities, the VS, VR, and OB groups seem to have their own identity.

The light green color means that the lipid species were identified in the respective group, while the orange color indicates absent species in the respective group. Samples were obtained from six olive orchards, located in Vilar Seco_1 (VS_1), Vilar Seco_2 (VS_2), Vilar Seco_3 (VS_3), Silgueiros (Sil), Oliveira de Barreiros (OB), and Vila Ruiva (VR).

The ANOSIM also found significant differences in the polar lipidomes of the six groups, showing R values ranging from 0.54 to 1.0 and *p* values < 0.05 (Supplementary Material, Table S5). Furthermore, statistical data revealed that the polar lipid fingerprint of olive seeds from different olive groves in the same location (Vilar Seco) is significantly different (Supplementary Material, Table S5).

**Figure 3:**
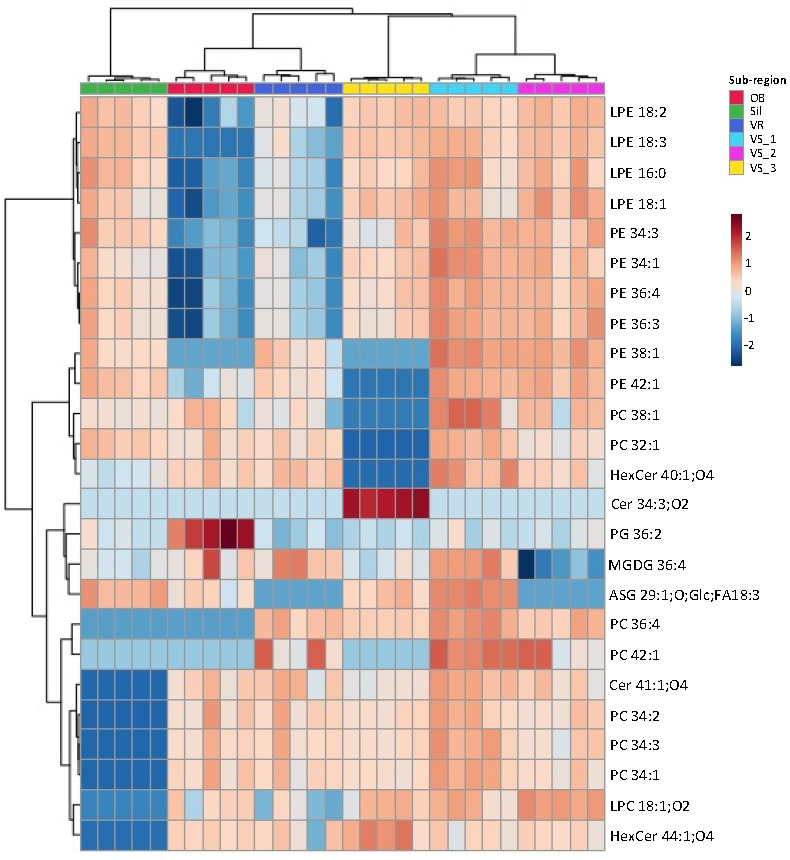
Hierarchical clustering heatmap of the polar lipid species data. Heatmap/clustering of the top 25 polar lipid species with lowest *q* values (i.e., the polar lipid species with the higher discriminating power). The dendrogram of the top represents the sample groups. The color scale shows the relative abundance levels, i.e., normalized peak areas, and the numbers indicate the fold difference from the mean. Lipid species abbreviation follows the LIPID MAPS nomenclature and shorthand notation, as C-atoms: number of double bond equivalents (DBE) and C-atoms: DBE; O-atoms for oxygenated lipids. Samples were collected in Nelas (Portugal) from six olive orchards located in Vilar Seco_1 (VS_1), Vilar Seco_2 (VS_2), Vilar Seco_3 (VS_3), Silgueiros (Sil), Oliveira de Barreiros (OB), and Vila Ruiva (VR).

The dataset was sorted to show more evident discrimination of these samples, using the lowest *q* values, and the top 25 lipid species with the lowest *q* values were ranked and used to create the heatmap (Figure 3). The list of 25 lipid species included seven PC, six PE, four LPE, two HexCer, two Cer, one PG, one MGDG, one LPC, and one ASG. Among the most discriminating species, 19 were PL (LPE, PE, PC, and PG and LPC). The grouping of these main lipid species that best explains the differences observed for the geographic identity of olive seeds showed discrimination between the six groups, with a close association of samples from the same location (Figure 3). The dendrogram at the top of the heatmap evidenced the first level of separation between the Sil group and the remaining groups (Figure 3). The second level of separation divided the OB and VR groups, which were clustered, from VS groups (VS_3, VS_1, and VS_2). The VS_1 and VS_2 groups also clustered together (Figure 3). In the Sil group, the species that contributed the most to the discrimination were Cer 41:1;O4, PC 34:2, PC 34:1, PC3 4:3, HexCer 44:1;O4 and LPC18:1;O2, which exhibited a lower normalized XIC area in these samples. It was, thus, possible to observe a characteristic polar lipid pattern for each group (Figure 3).

**Figure 4:**
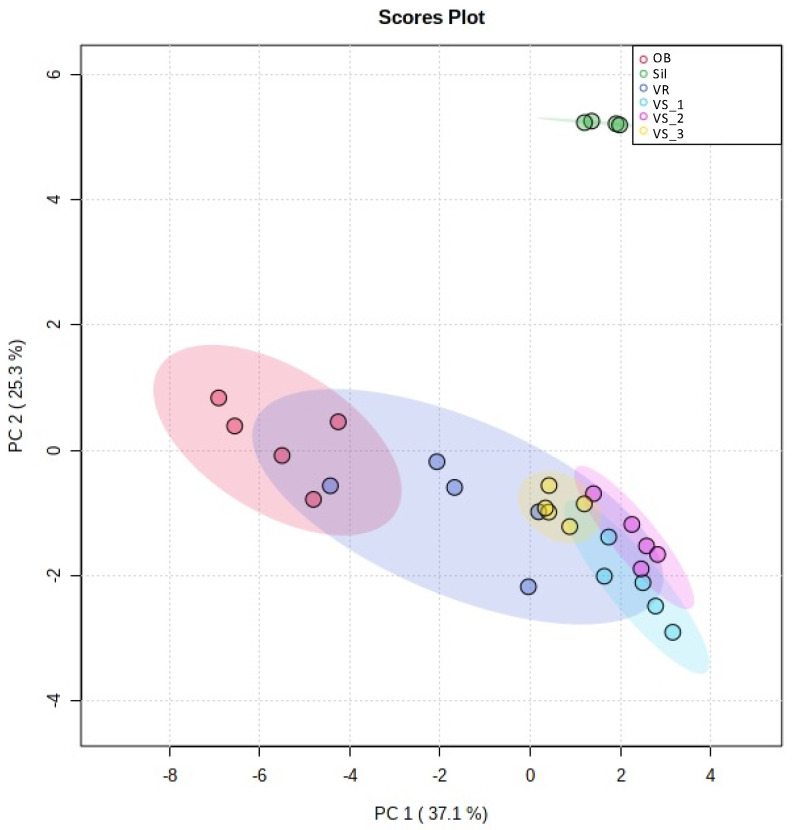
Principal component analysis (PCA) scores plot of the polar lipid species displaying the lowest *q* values, i.e., the top 25 polar lipid species with the higher discriminating power. Sub-region abbreviations: Vilar Seco_1 (VS_1), Vilar Seco_2 (VS_2), Vilar Seco_3 (VS_3), Silgueiros (Sil), Oliveira de Barreiros (OB), and Vila Ruiva (VR).

To show the most remarkable differences between the six sub-regions, a new PCA was performed with the 25 lipid species that most contributed to the discrimination between the sampling sites (Figure 4). An increase in the PC 1 plus PC 2 eigenvalues explained a total variance of 62.4% of the observations. PCA analysis of the 25 most discriminating lipid species in the LC-MS dataset revealed six groups representing each location (Figure 4). The Sil group appeared isolated from the other groups, in opposition to the three VS groups at PC 2 and the OB group at PC 1. The five groups, excluding Sil, were located mainly in negative values of PC 2 with some overlaps. The three VS groups (VS_1, VS_2, and VS_3) appeared very close, showing some overlap, even though there was some independence. On the other hand, the OB group also stood out from these three groups and from the Sil group. The VR group exhibited the most remarkable heterogeneity variance, partially overlapping with the OB group in negative values of PC 1 and VS groups in positive values of PC 1 (Figure 4).

**Figure 5:**
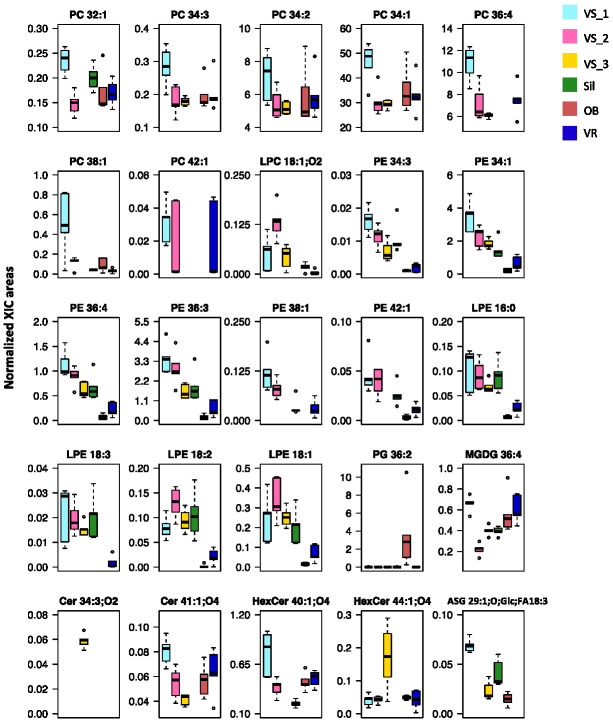
Boxplots of the top 25 polar lipid species sorted by the lowest *q* values. Normalized areas were obtained from extracted-ion chromatograms (XIC). The boxes represent medians and 25th and 75th percentiles. Error bars represent range (from the minimum to the maximum value), and dots are outliers. Sub-region abbreviations: Oliveira de Barreiros (OB), Silgueiros (Sil), Vila Ruiva (VR), Vilar Seco_1 (VS_1), Vilar Seco_2 (VS_2), and Vilar Seco_3 (VS_3).

The normalized XIC areas of these 25 most discriminating lipid species are illustrated in Figure 5. Their contribution to sub-geographic discrimination was assessed using a pairwise univariate comparison (Supplementary Material, Table S6). The significance levels in the variability allowed us to identify specific compounds as prospective candidates to discriminate the geographic origin of olive seeds. Sil samples were explicitly attributed to this location, through the absence of Cer 41:1;O4, PC 34:2, PC 34:3, PC 34:1, LPC 18:1;O2 and HexCer 44:1;O4 (Figure 5 and Table 1). The olive seeds from the OB group were distinguished by their high normalized XIC areas in PG 36:2 and lower normalized XIC areas, or absence of some LPE and PE species. The specificity of geographic origin of the samples from the VR group was revealed by the high values of the normalized XIC areas of PC 42:1 and MGDG 36:4, absence of ASG 29:1;O;Glc;FA18:3, and lower values of the normalized XIC areas of LPE and PE. Although similar to each other, the samples from Vilar Seco showed significant differences, which allowed the VS_3 group to stand out from VS_1 and VS_2, by the absence of PE 38:1, PE 42:1, PC 38:1, PC 32:1 and HexCer 40:1;O4, and by the high relative abundance in Cer 34:3;O2. The VS_1 and VS_2 groups showed greater similarities and stood out from the other groups due to the normalized XIC areas of LPE, PE, and PC lipid species.

Univariate analysis data (Supplementary Material, Table S6) of the polar lipid profile of the six groups of olive seeds revealed unique profiles. Each group has a molecular identity, which, in this case, depends on the sub-geographical origin.

## 4. Discussion

This study aimed to provide a thorough characterization of the polar lipidome of olive seeds on the Portuguese cultivar *Galega vulgar*, to provide new insight into their nutritional value, potential health benefits, and chemical identity related to the geographic origin, using an untargeted LC-MS/MS analysis.

### 4.1. Oil Yield and Polar Lipid Content in Olive Seeds

Olive seeds had an average of 25.6% of total lipids. The total average of PL concentration was 1.25%, and the total average concentration of GL was 1.49%. The PL/GL ratio was equal to 1.0, on average. The Sil and OB groups had more GL than PL (PL/GL ratio less than 1.0), while the VS and VR groups had more PL than GL (ratio greater than 1.0), but the differences were not significant. Therefore, statistical differences between the six groups were only observed for the total PL content.

Previous studies have found 30–43% lipids in olive seeds of other varieties of olives, cultivated at different latitudes [2,3] and 0.1% PL in cv. *Chemlal* from Algeria [3]. Variations in the oil yield could be assigned to the olive variety, for example, since the pulp/stone ratio is quite different between varieties and to several agronomic and edaphoclimatic factors [13]. Nevertheless, we found a PL concentration ten-fold higher than that reported by Moussaoui et al. (2008) [3]. This could be due to agronomic and edaphoclimatic factors, as well as the methods for extracting total lipids and quantifying PL, which were different from those used herein. To our knowledge, no study has reported the GL content or differences in the total lipid, PL, and GL content in olive seeds from different geographic origins.

Thus, the results showed that olive seeds are a good source of oil (over 25%), with a high amount of polar lipids (ca. 2.74%, on average) compared to other usually valued minor components, such as phenolic compounds, sterols, or tocopherols. Polar lipids are bioactive phytochemicals that add value to these fruit seeds, with a wide range of applications in different industries, such as food, nutraceuticals, feed, and cosmetics [10].

### 4.2. Polar Lipid Fingerprint of Olive Seeds

The polar lipidome of the olive seeds from four sub-regions of Nelas (Portugal) comprised PL (PC, LPC, PE, LPE, PG), GL (MGDG and DGDG), SL (Cer and HexCer), and sterol derivatives (ASG). The fatty acyl chains ranged from 16:0 to 24:0, up to three double bonds, and some oxygenated species in the PC, LPC, Cer, and HexCer classes.

Montealegre et al. (2013) analyzed the composition of PL in seeds and pulp of olive fruits of different Spanish cultivars, using non-aqueous capillary electrophoresis coupled to ESI-MS [12]. The authors found PA (88%), PI (6%), PC (4.4%), PG (1.2%), and lysoPA (0.2%) in olive seeds cv. *Arbequina* [12]. Fifteen molecular species were identified, but several lipid categories were overlooked (GL, SL, or sterol derivatives).

The polar lipidome of the olive seeds is remarkably different from the pulp. The pulp has a greater diversity of PC and LPC species [15], with FA chains ranging from 8:1 to 25:0, including several unusual odd, polyunsaturated, and short chains [15]. Contrarily to what was observed for the olive pulp cv. *Galega vulgar*, we have found neither sphingomyelin nor betaine lipids in olive seeds. Nevertheless, we found LPE, Cer, and ASG in the seeds, which were not detected in the pulp of the same samples [15]. The oxPC, oxLPC, and HexCer[AH] species found in these seeds have also been reported in the olive pulp in previous work by our group [15].

In preliminary studies on olive seeds cv. *Galega vulgar*, we used normal-phase SPE silica columns for recovering polar lipids, TLC for class separation, and direct ESI-MS/MS of TLC spots to identify the lipid classes and molecular species [14]. In SPE, the polar lipids were eluted with acetone and then with MeOH. As a result, PC, LPC, PE, LPE, and PG could be recovered, as in the present study, as well as other PL, including PI, PA, *N*-acyl-PE, and other GL, such as sulfoquinovosyldiacylglycerol (SQDG) [14], which were not detected in our previous study. However, at that time, we could not identify Cer, HexCer, or ASG, neither in the SPE fraction nor in the TLC spots.

The systematic polar lipid fingerprint in olives (seed and pulp), olive oils, and olive pomace is important, because the polar lipid fraction of olive oil and olive pomace were reported to have beneficial properties for health, such as antithrombotic and anti-atherosclerotic activity in vitro and in vivo in rabbits [7,8,9,11]. Furthermore, bioactive glycerylether-*sn*-2-acetyl GL with anti-PAF activity was found in olive pomace [11]. Therefore, further studies are needed to improve the complete recovery of the polar lipidome from olive seed oil to include anionic polar lipids (e.g., PA, PG, PI, and SQDG) and enable a comprehensive LC-MS/MS polar lipid phenotyping for further bioprospection. Likewise, they are sources of n-6 and n-3 essential fatty acids.

In addition, the polar lipidome of olive seeds revealed significant differences between the six groups under study. These differences occurred in the relative abundance of lipid classes, the presence or absence of specific lipid species, changes in the degree of unsaturation of the lipid species in lipid categories, and differences in total PL content. In plants, the plasticity of the polar lipid profile is generally associated with various types of biotic (e.g., pathogen infections or attack by predators and parasites) and abiotic stress (e.g., cold, heat, drought, access to water, soil composition) [28]. When the soil is deficient in phosphorus, the proportion of GL, major constituents of chloroplasts and essential for normal plant growth, generally increases compared to PL [29]. The variation in the degree of unsaturation and fatty acid composition of plant tissues also varies with climatic and culture conditions and the stage of tissue development [28]. Besides, oxygenated PL (oxPC and oxLPC) were identified. The formation of enzymatically oxidized PL results from various stress factors, such as response to temperature extremes, light intensity, and UV-B radiation [30], can have profound biological effects on animals and plants [31]. On the other hand, HexCer exists in photosynthetic plant tissues, namely HexCer with 2-hydroxy fatty acids, which corroborates our results. These lipids help the plasma membrane to cope with stressful situations, such as cold, drought, and attacks by fungal pathogens [32]. The concentration of species with 2-hydroxy long-chain fatty acids increases in plants that are more tolerant to cooling and freezing. HexCer are vital components of the human skin, as they maintain the water permeability barrier, but they are also found in neurons and several fungal pathogens [32].

Although our sampling was limited to a specific region (Nelas), there are considerable annual thermal amplitudes and altitude variations at this location. Thus, for example, Silgueiros stood out from the other five sample groups in the PCA analysis (Figure 4). Silgueiros is located at the lowest altitude (303 m) compared to the other five locations (over 390 m), (Supplementary Material, Table S7). The average climatological data available from the regions of Viseu Dão-Lafões, where the locality of Nelas is situated, does not allow us to reveal specific climatic differences between the different sampling locations (Supplementary Material, Table S7). However, differences in elevation, climatic conditions, and soil composition certainly contributed to the observed differences.

In summary, the six groups of samples shared the same families and classes of polar lipids, but showed variations within each class between groups, depending on the edaphoclimatic and stress factors to which each olive grove was subjected, demonstrating that each group has a unique fingerprint.

### 4.3. Usefulness of Lipidomics Data for Comparing Sub-Geographic Locations

Polar lipids have also shown great potential to be markers of authenticity, detect fraud and assign a molecular fingerprint to different categories of olive oil [33,34]. Therefore, in this study, we aimed to verify whether there is a cultivar-specific polar lipid fingerprint or whether the geographic sub-region affects the polar lipid profile of olive seeds.

The first multivariate analysis of the lipidomic data showed unclear discrimination of the sub-geographic regions (Supplementary Material, Figure S8). However, after considering only the 25 lipid species that contributed the most to the discrimination, there was a more apparent distinction between the samples from the six olive groves. Despite the proximity of groups, from the same geographic region and from the same botanical variety, certain LPE, PE, and PC species can be highlighted as lipid markers to discriminate between groups. The OB and VR groups had a significant decrease in these LPE and PE species, unlike the VS_1 and VS_2 groups, in which, in addition to these two classes, there was also an increase in the relative abundance of PC. The univariate data analysis (Figure 5) showed the most remarkable differences observed in the most discriminating lipid species between groups. In the Sil group, those were the 34-carbon PC and the absence of LPC 18:1;O2. In the OB and VR groups, there were remarkably low abundances of LPE and PE. Furthermore, in VS_1 and VS_2, the relative abundance of all the 25 lipid species was high, except for one Cer and one PG.

Interestingly, the most discriminating lipid species are PL, which may indicate greater plasticity in membrane composition due to the ripeness stage of the fruit and biotic stress factors. Differences in the abundance of the lyso forms in specific locations may be related to changes in environmental conditions. Lysophospholipids are formed by hydrolysis of the fatty acid chain of PL. Lysophospholipids are very sensitive to environmental stresses, such as heat shock, cold, and freezing [35]. The levels of LPE and LPC change significantly during the hydration–dehydration cycles on seeds of *Arabidopsis thaliana*, *Lolium perenne*, *Nicotiana tabacum* [36]. LPE decreases during dehydration and increases during hydration. Moreover, the content of membrane lipids, such as MDGD, DGDG, PC, PE, PI, PS, and PG increased and decreased markedly, as moisture varied during the hydration and dehydration of those seeds [36].

Although the geographical distance between the olive groves is very short, the PCA allowed us to cluster each group individually and identify the main lipids that can be used as identity markers. Thus, multivariate statistical analysis of the polar lipidome data showed that it is possible to identify unique polar lipid profiles, in independent groups of olive seeds of the same olive variety and the same geographic region. On the one hand, it was found that groups from the same location (Vilar Seco) have their unique profiles, with different families of polar lipids contributing to discrimination. On the other hand, when groups of samples from different sub-regions (six olive groves from four localities) were compared, it was evident that PL but also GL, SL, and ASG are important markers of discrimination.

Phospholipidomics has recently been used as a promising strategy to classify olive oils and find chemical quality markers [34,37]. Virgin and extra virgin olive oils have a unique PC profile, and GL are equally significant in assigning a molecular fingerprint to each olive oil [33]. Furthermore, a targeted LC-MS/MS analysis, comparing the phospholipidome of different categories of olive oil, showed that the molecular species of PA and PG are crucial for discrimination [34].

On the contrary, SL or GL were generally excluded from the analyses. However, GL can make a difference in assigning a molecular fingerprint to each olive oil [33], and the bioactive compound found in the polar lipid fraction of olive pomace was a GL, as mentioned above. Furthermore, untargeted lipidomics approaches have the advantage of fully covering the lipidome, to include other lipid classes that have generally not yet been identified in these matrices, and have allowed the discovery of new molecules that may be essential for the characterization of olive samples from different origins, and the bioprospection of bioactive polar lipids [15,33]. Different compositions may be important for identifying bioactive compounds with greater nutritional value, which may, thus, contribute to the valorization of olive seed oils from specific regions or with edaphoclimatic traits. Olive seed flour and oil are already marketed for the food and cosmetic industries but on a microscale [1].

Therefore, lipidomic data are essential for assigning a fingerprint to olive seeds from different geographic sub-regions. All classes of polar lipids are relevant for this differentiation, with a more notable contribution from LPE, PE, and PC.

## 5. Conclusions

The polar lipid fingerprinting of olive seeds cv. *Galega vulgar,* by HILIC-LC-HR-ESI-HRMS and MS/MS, allowed the identification of almost a hundred lipid species, from ten different classes of PL, GL, SL, and sterol derivatives. Some of them have been described as having biological activity, which will bring a new vision on their role in the health benefits of olive seeds as functional ingredients. Therefore, a broader understanding of the chemical composition of olive seeds is key for the bioprospection of bioactive polar lipids. The lipidomic approach also enabled the distinguishing of sub-geographic locations, revealing for the first time a site-specific chemical fingerprint based on polar lipid markers. Although composed of the same lipid classes, each location demonstrated a unique polar lipid profile of interest for identity, quality, and authenticity. Further studies with samples of olives from other varieties and regions will be fundamental to identify target lipid compounds that are important in classifying olives and olive oils.

## Figures and Tables

**Table 1:** Missing species of polar lipids identified in the olive seeds cv. *Galega vulgar* from different sub-regions of Nelas, Portugal.

Sample Group	VS_1	VS_2	VS_3	Sil	OB	VR
PC 32:1						
PC 34:3						
PC 34:2						
PC 34:1						
PC 36:4						
PC 36:4;O2						
PC 38:1						
PC 36:2;O2						
PC 41:1						
PC 42:1						
LPC 18:1;O2						
PE 38:1						
PE 42:1						
LPE 18:3						
PG 36:1						
DGDG 34:3						
DGDG 36:6						
Cer 34:3;O2						
Cer 41:1;O4						
HexCer 40:1;O4						
HexCer 44:1;O4						
ASG 29:1;O;Glc;FA18:3						
**total missing species**	**2**	**4**	**8**	**11**	**7**	**2**

## Data Availability

Data is contained within the article (or supplementary material).

## Supplementary Materials

The following supporting information can be downloaded at: https://www.mdpi.com/article/10.3390/foods11070951/s1, Figure S1: Sampling locations (green circle) for Galega vulgar cv. olives in traditional olive groves under rainfed conditions in Nelas (Portugal) in the 2016/2017 campaign. Five biological replicates of olive seeds were used in this study for each sample group; Table S1: Sampling locations for olives cv. Galega vulgar in traditional olive groves under rainfed conditions in Nelas, Portugal, in the 2016/2017 campaign; Table S2: Content of total lipids, phospholipids (PL) and glycolipids (GL) in olive (Olea europaea L. cv. Galega vulgar) seeds from different sub-regions of Nelas (Portugal); Table S3: List of polar lipids (phospholipids, glycolipids, sphingolipids and acyl sterol glycosides) identified in the olive (Olea europaea L. cv. Galega vulgar) seeds by HILIC-LC-ESI-MS and MS/MS; Table S4: Total number of lipid species by polar lipid category and number of lipid species by polar lipid class identified in the olive seeds of the different sub-regions of Nelas, Portugal. Samples were collected in Nelas (Portugal) from six olive groves; Figure S2: Illustrative LC-MS/MS spectra of the phosphatidylcholine and lysophosphatidylcholine classes identified in the olive seed cv. Galega vulgar. PC 36:3;O at m/z 800.58 as [M + H]+ (A) and at m/z 858.59 as [M + CH3COO]− (B). LPC 18:2 at m/z 520.34 as [M + H]+ (C) and at m/z 578.35 as [M + CH3COO]− (D). The notation C:DBE;O of the lipid species means the total number of carbon atoms (C), double bond equivalents (DBE), and the number of oxygen atoms (O). A general chemical structure of each class is also shown; Figure S3: Illustrative LC-MS/MS spectra of the phosphatidylethanolamine and lysophosphatidylethanolamine classes identified in the olive seed cv. Galega vulgar. PE 42:1 at m/z 830.67 as [M + H]+ (A) and at m/z 828.65 as [M − H]− (B). LPE 18:1 at m/z 480.31 as [M + H]+ (C) and at m/z 478.30 as [M − H]− (D); Figure S4: Illustrative LC-MS/MS spectrum of the phosphatidylglycerol class identified in the olive seed cv. Galega vulgar, PG 36:2 at m/z 773.53 as [M − H]-; Figure S5: Illustrative LC-MS/MS spectra of the glyceroglycolipid classes identified in the olive seed cv. Galega vulgar: monogalactosyldiacylglycerol MGDG 36:4 at m/z 796.60 as [M + NH4]+ (A) and digalactosyldiacylglycerol DGDG 34:1 at m/z 936.66 as [M + NH4]+ (B); Figure S6: Illustrative LC-MS/MS spectra of the sphingolipid classes identified in the olive seed cv. Galega vulgar: Ceramide Cer 42:1;O4 at m/z 682.49 as [M + H]+ (A) and hexosylceramide HexCer 38:1;O4 at m/z 788.63 as [M + H]+ (B). The notation C:DBE;O of the lipid species means the total number of carbon atoms (C), double bond equivalents (DBE), and the number of oxygen atoms (O); Figure S7: Illustrative LC-MS/MS spectrum of an acyl sterol glycoside identified in the olive seed cv. Galega vulgar, 18:2-Glc-Sitosterol at m/z 856.71 as [M + NH4]+; Figure S8: Principal components analysis (PCA) scores plot of the two first PC (PC2 vs. PC1) (A), PCA loadings plot (B), and PCA biplot (C) performed on the whole standardized and log-transformed polar lipid species data set acquired by HILIC-LC-MS of olive seeds cv. Galega vulgar from different sub-regions of Nelas (Portugal): Vilar Seco_1 (VS_1), Vilar Seco_2 (VS_2), Vilar Seco_3 (VS_3), Silgueiros (Sil), Oliveira de Barreiros (OB), and Vila Ruiva (VR); Table S5: Summary of ANOSIM analysis comparing the polar lipid profiles of olive seeds cv. Galega vulgar from different sub-regions of Nelas (Portugal): Vilar Seco_1 (VS_1), Vilar Seco_2 (VS_2), Vilar Seco_3 (VS_3), Silgueiros (Sil), Oliveira de Barreiros (OB), and Vila Ruiva (VR); Table S6: One-way analysis of variance (ANOVA) of the glog transformed and autoscaled HILIC-LC-MS data of polar lipid molecular species from olive seeds cv. Galega vulgar, followed by post-hoc Tukey’s multiple comparison test and p-values correction for multiple testing using Benjamini–Hochberg false discovery rate (FDR, q values). Samples were collected in Nelas (Portugal) from six olive orchards located in Vilar Seco_1 (VS_1), Vilar Seco_2 (VS_2), Vilar Seco_3 (VS_3), Silgueiros (Sil), Oliveira de Barreiros (OB), and Vila Ruiva (VR); Table S7: Geographical and geological data of the studied sub-regions of Nelas (Portugal) and average climatological data of the Viseu Dão-Lafões regions where the locality of Nelas belongs.
