# Supplementary material for: Bioprospecting Bioactive Polar Lipids from Olive (Olea europaea cv. Galega vulgar) Fruit Seeds: LC-HR-MS/MS Fingerprinting and Sub-Geographic Comparison
Source: Foods. 2022 Mar 25;11(7):951. doi: 10.3390/foods11070951 (PMC8997722; doi:10.3390/foods11070951)
Supplement: Supplementary file 1 [file foods-11-00951-s001.zip › foods-1572716-supplementary.pdf]

Supplementary Materials for

Bioprospecting Bioactive Polar Lipids from Olive (*Olea europaea* cv. *Galega vulgar*) Fruit Seeds: LC-HR-MS/MS Fingerprinting and Sub-Geographic Comparison

Eliana Alves [1,*], Felisa Rey [2], Tânia Melo [1,2], Madalena P. Barros [3], Pedro Domingues [1] and Rosário Domingues [1,2]

[1] Mass Spectrometry Centre, LAQV-REQUIMTE & Department of Chemistry, University of Aveiro, Campus Universitário de Santiago, 3810-193 Aveiro, Portugal

[2] ECOMARE & CESAM - Centre for Environmental and Marine Studies, Department of Chemistry, University of Aveiro, Campus Universitário de Santiago, 3810-193 Aveiro, Portugal

[3] Cooperativa de Olivicultores de Nelas, C.R.L., Zona Industrial de Nelas, 3520-095 Nelas, Portugal

* Corresponding author: elianaalves@ua.pt (E.A.)

Contents

Figure S1. Sampling locations (green circle) for *Galega vulgar* cv. olives in traditional olive groves under rainfed conditions in Nelas (Portugal) in the 2016/2017 campaign. Five biological replicates of olive seeds were used in this study of each sample group.. 3

Table S1. Sampling locations for olives cv. *Galega vulgar* in traditional olive groves under rainfed conditions in Nelas, Portugal, in the 2016/2017 campaign....................... 3

Table S2. Content of total lipids, phospholipids (PL) and glycolipids (GL) in olive (*Olea europaea* L. cv. *Galega vulgar*) seeds from different sub-regions of Nelas (Portugal)... 4

Table S3. List of polar lipids (phospholipids, glycolipids, sphingolipids and acylsterolglycosides) identified in the olive (*Olea europaea* L. cv. *Galega vulgar*) seeds by HILIC-LC-ESI-MS and MS/MS. 5

Table S4. Total number of lipid species by polar lipid category and number of lipid species by polar lipid class identified in the olive seeds of the different sub-regions of Nelas, Portugal. Samples were collected in Nelas (Portugal) from six olive groves. 8

Figure S2. Illustrative LC-MS/MS spectra of the phosphatidylcholine and lysophosphatidylcholine classes identified in the olive seed cv. *Galega vulgar*. PC 36:3;O at *m/z* 800.58 as $[M + H]^+$ (A) and at *m/z* 858.59 as $[M + CH_3COO]^-$ (B). LPC 18:2 at *m/z* 520.34 as $[M + H]^+$ (C) and at *m/z* 578.35 as $[M + CH_3COO]^-$ (D). The notation C:DBE;O of the lipid species means the total number of carbon atoms (C), double bond

equivalents (DBE), and the number of oxygen atoms (O). A general chemical structure of each class is also shown. 9

Figure S3. Illustrative LC-MS/MS spectra of the phosphatidylethanolamine and lysophosphatidylethanolamine classes identified in the olive seed cv. *Galega vulgar*. PE 42:1 at *m/z* 830.67 as $[M + H]^+$ (A) and at *m/z* 828.65 as $[M - H]^-$ (B). LPE 18:1 at *m/z* 480.31 as $[M + H]^+$ (C) and at *m/z* 478.30 as $[M - H]^-$ (D)... 10

Figure S4. Illustrative LC-MS/MS spectrum of the phosphatidylglycerol class identified in the olive seed cv. *Galega vulgar*, PG 36:2 at *m/z* 773.53 as $[M - H]^-$........................ 11

Figure S5. Illustrative LC-MS/MS spectra of the glyceroglycolipid classes identified in the olive seed cv. *Galega vulgar*: monoglycosyldiacylglycerol MGDG 36:4 at *m/z* 796.60 as $[M + NH_4]^+$ (A) and diglycosyldiacylglycerol DGDG 34:1 at *m/z* 936.66 as $[M + NH_4]^+$ (B).. 12

Figure S6. Illustrative LC-MS/MS spectra of the sphingolipid classes identified in the olive seed cv. *Galega vulgar*: Ceramide Cer 42:1;O4 at *m/z* 682.49 as $[M + H]^+$ (A) and hexosylceramide HexCer 38:1;O4 at *m/z* 788.63 as $[M + H]^+$ (B). The notation C:DBE;O of the lipid species means the total number of carbon atoms (C), double bond equivalents (DBE), and the number of oxygen atoms (O). 13

Figure S7. Illustrative LC-MS/MS spectrum of an acylsterolglycoside identified in the olive seed cv. *Galega vulgar*, 18:2-Glc-Sitosterol at *m/z* 856.71as $[M + NH_4]^+$. 14

Figure S8. Principal components analysis (PCA) scores plot of the two first PC (PC2 versus PC1) (A), PCA loadings plot (B), and PCA biplot (C) performed on the whole standardized and log-transformed polar lipid species data set acquired by HILIC-LC-MS of olive seeds cv. *Galega vulgar* from different sub-regions of Nelas (Portugal): Vilar Seco_1 (VS_1), Vilar Seco_2 (VS_2), Vilar Seco_3 (VS_3), Silgueiros (Sil), Oliveira de Barreiros (OB), and Vila Ruiva (VR).. 15

Table S5. Summary of ANOSIM analysis comparing the polar lipid profiles of olive seeds cv. *Galega vulgar* from different sub-regions of Nelas (Portugal): Vilar Seco_1 (VS_1), Vilar Seco_2 (VS_2), Vilar Seco_3 (VS_3), Silgueiros (Sil), Oliveira de Barreiros (OB), and Vila Ruiva (VR).. 16

Table S6. One-way analysis of variance (ANOVA) of the glog transformed and autoscaled HILIC-LC-MS data of polar lipid molecular species from olive seeds cv. *Galega vulgar*, followed by post-hoc Tukey's multiple comparison test and *p*-values correction for multiple testing using Benjamini–Hochberg false discovery rate (FDR, q values). Samples were collected in Nelas (Portugal) from six olive orchards located in Vilar Seco_1 (VS_1), Vilar Seco_2 (VS_2), Vilar Seco_3 (VS_3), Silgueiros (Sil), Oliveira de Barreiros (OB), and Vila Ruiva (VR)... 17

Table S7. Geographical and geological data of the studied sub-regions of Nelas (Portugal) and average climatological data of the Viseu Dão-Lafões regions where the locality of Nelas belongs.. 19

Reference 20

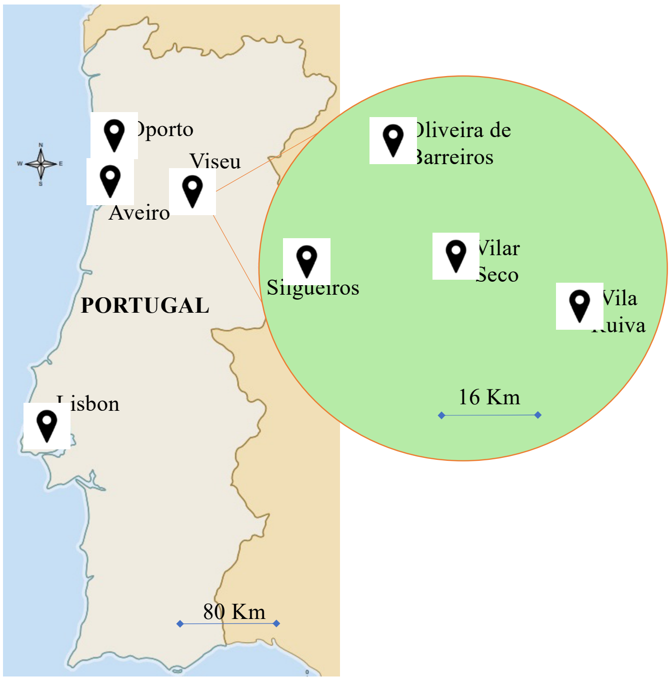

Figure S1. Sampling locations (green circle) for *Galega vulgar* cv. olives in traditional olive groves under rainfed conditions in Nelas (Portugal) in the 2016/2017 campaign. Five biological replicates of olive seeds were used in this study of each sample group.

Table S1. Sampling locations for olives cv. *Galega vulgar* in traditional olive groves under rainfed conditions in Nelas, Portugal, in the 2016/2017 campaign.

Region	Vilar Seco			Silgueiros	Oliveira de Barreiros	Vila Ruiva
Olive grove	#1	#2	#3	#4	#5	#6
Group name	VS_1	VS_2	VS_3	Sil	OB	VR

Table S2. Content of total lipids, phospholipids (PL) and glycolipids (GL) in olive (*Olea europaea* L. cv. *Galega vulgar*) seeds from different sub-regions of Nelas (Portugal).

Sample group	Biological replicates	Olive seed mass (mg)	Gravimetric extraction yield (mg)	Gravimetric extraction yield (%)	PL in the total lipid extract (µg·mg^{-1})	GL in the total lipid extract (µg·mg^{-1})	PL + GL content in the total lipid extract (µg·mg^{-1})	PL/GL ratio
**Vilar Seco_1 (VS_1)**	Average	313.4	79.4	25.4	15.78 [a]	13.87	29.65	1.25
	S.D.	10.61	3.90	1.44	3.89	3.95	4.17	0.49
**Vilar Seco_2 (VS_2)**	Average	303.4	78.8	26.0	15.09 [a]	14.70	29.79	1.20
	S.D.	3.08	3.54	0.91	3.13	6.17	6.40	0.54
**Vilar Seco_3 (VS_3)**	Average	308.7	77.7	25.2	12.94 [a,b]	14.23	27.18	1.03
	S.D.	1.38	4.06	1.34	1.84	5.42	6.31	0.32
**Silgueiros (Sil)**	Average	208.8	53.1	25.4	10.61 [a,b]	20.52	31.13	0.59
	S.D.	10.33	3.01	1.11	1.89	6.68	6.12	0.25
**Oliveira de Barreiros (OB)**	Average	307.6	81.4	26.5	11.31 [a,b]	10.14	21.44	1.21
	S.D.	5.01	3.84	1.39	1.68	2.52	1.82	0.40
**Vila Ruiva (VR)**	Average	309.7	78.3	25.3	9.56 [b]	15.65	25.21	0.63
	S.D.	4.83	2.45	1.16	0.97	2.71	2.79	0.15
**Total average**		291.9	74.8	25.6	12.5	14.9	27.4	1.0

Different superscript letters indicate significant differences between groups (one-way ANOVA, $p < 0.05$). S.D., standard deviation.

Table S3. List of polar lipids (phospholipids, glycolipids, sphingolipids and acylsterolglycosides) identified in the olive (*Olea europaea* L. cv. *Galega vulgar*) seeds by HILIC-LC-ESI-MS and MS/MS.

Lipid species	t_R	Observed *m/z*	Calculated *m/z*	Error (ppm)	Fatty acyl chains	Formula
**PC identified as [M+H]$^+$**						
PC 32:1	11.81	732.5567	732.5543	3.28	a)	C40H79NO8P
PC 33:1	11.90	746.5714	746.5700	1.88	a)	C41H81NO8P
PC 34:4	11.92	754.5382	754.5387	-0.66	a)	C42H77NO8P
PC 34:3	11.70	756.5579	756.5543	4.76	a)	C42H79NO8P
PC 34:2	11.70	758.5729	758.5700	3.82	18:2/16:0 and 18:1/16:1	C42H81NO8P
PC 34:1	11.70	760.5884	760.5856	3.68	18:1/16:0	C42H83NO8P
PC 35:3	11.70	770.5718	770.5700	2.34	a)	C43H81NO8P
PC 35:2	11.70	772.5859	772.5856	0.39	18:1/17:1	C43H83NO8P
PC 34:2;O	11.70	774.5675	774.5649	3.36	a)	C42H81NO9P
PC 35:1	11.19	774.6040	774.6013	3.49	a)	C43H85NO8P
PC 36:6	11.70	778.5411	778.5387	3.08	a)	C44H77NO8P
PC 36:5	11.70	780.5553	780.5543	1.28	a)	C44H79NO8P
PC 36:4	11.73	782.5709	782.5670	4.98	18:2/18:2 and 18:1/18:3	C44H81NO8P
PC 36:3	11.19	784.5886	784.5856	3.82	18:1/18:2	C44H83NO8P
PC 36:2	11.19	786.6042	786.6013	3.69	18:1/18:1 and 18:2/18:0	C44H85NO8P
PC 34:1;O2	10.66	792.5732	792.5755	-2.90	a)	C42H83NO10P
PC 36:4;O	11.70	798.5655	798.5649	0.75	a)	C44H81NO9P
PC 36:3;O	11.70	800.5825	800.5805	2.50	18:1/18:2;O	C44H83NO9P
PC 37:2	11.19	800.6178	800.6169	1.12	a)	C45H87NO8P
PC 36:2;O	11.70	802.5978	802.5962	1.99	18:1/18:1;O	C44H85NO9P
PC 38:3	11.19	812.6161	812.6169	-0.98	a)	C46H87NO8P
PC 38:2	11.19	814.6351	814.6326	3.07	18:1/20:1	C46H89NO8P
PC 36:4;O2	11.02	814.5633	814.5598	4.30	a)	C44H81NO10P
PC 38:1	11.22	816.6509	816.6482	3.31	18:1/20:0	C46H91NO8P
PC 36:3;O2	10.32	816.5763	816.5755	0.98	a)	C44H83NO10P
PC 36:2;O2	10.72	818.5942	818.5911	3.79	a)	C44H85NO10P
PC 41:1	9.84	858.6933	858.6952	-2.19	a)	C49H97NO8P
PC 42:1	10.86	872.7149	872.7108	4.70	a)	C50H99NO8P
**LPC identified as [M+H]$^+$**						
LPC 16:0	14.79	496.3421	496.3403	3.63	16:0	C24H51NO7P
LPC 18:3	15.19	518.3247	518.3247	0.00	a)	C26H49NO7P
LPC 18:2	15.19	520.3426	520.3403	4.42	18:2	C26H51NO7P
LPC 18:1	14.87	522.3581	522.3560	4.02	18:1	C26H53NO7P
LPC 20:1	14.87	550.3894	550.3873	3.88	a)	C28H57NO7P
LPC 20:0	14.62	552.4047	552.4029	3.23	a)	C28H59NO7P
LPC 18:1;O2	14.85	554.3478	554.3458	3.61	a)	C26H53NO9P
LPC 22:0	14.53	580.4348	580.4342	1.00	a)	C30H63NO7P
**PE identified as [M+H]$^+$**						
PE 30:3	4.65	658.4453	658.4448	0.79	a)	C35H65NO8P
PE 34:3	4.29	714.5097	714.5074	3.24	a)	C39H73NO8P
PE 34:2	4.16	716.5261	716.5230	4.28	18:2/16:0 and 18:1/16:1	C39H75NO8P
PE 34:1	4.16	718.5417	718.5387	4.18	18:1/16:0	C39H77NO8P
PE 36:5	4.16	738.5103	738.5074	3.95	a)	C41H73O8NP
PE 36:4	4.16	740.5252	740.5230	2.93	a)	C41H75NO8P
PE 36:3	4.16	742.5418	742.5387	4.17	18:1/18:2	C41H77NO8P
PE 36:2	4.16	744.5570	744.5543	3.63	18:1/18:1 and 18:2/18:0	C41H79NO8P
PE 36:1	4.02	746.5663	746.5700	-4.93	18:1/18:0	C41H81O8NP
PE 38:2	4.16	772.5881	772.5856	3.19	18:1/20:1	C43H83O8NP
PE 38:1	4.13	774.6034	774.6013	2.73	18:1/20:0	C43H85NO8P
PE 42:1	4.02	830.6655	830.6639	1.95	18:1/24:0	C47H93NO8P

**LPE identified as [M+H]$^+$**						
LPE 16:0	6.16	454.2947	454.2934	2.93	16:0	C21H45NO7P
LPE 18:3	6.16	476.2770	476.2777	-1.47	18:3	C23H43NO7P
LPE 18:2	6.16	478.2954	478.2934	4.25	18:2	C23H45NO7P
LPE 18:1	6.16	480.3111	480.3090	4.34	18:1	C23H47NO7P
**PG identified as [M-H]$^-$**						
PG 34:1	1.58	747.5179	747.5176	0.40	*	C40H76O10P
PG 36:3	1.84	771.5206	771.5176	3.89	*	C42H76O10P
PG 36:2	1.84	773.5360	773.5333	3.49	18:1/18:1	C42H78O10P
PG 36:1	1.77	775.5498	775.5489	1.16	*	C42H80O10P
**MGDG identified as [M+NH$_4$]$^+$**						
MGDG 34:2	2.08	772.5962	772.5939	3.01	18:2/16:0	C43H82NO10
MGDG 34:1	2.28	774.6108	774.6095	1.65	18:1/16:0	C43H84NO10
MGDG 36:6	2.11	792.5658	792.5626	4.04	18:3/18:3	C45H78NO10
MGDG 36:5	2.08	794.5801	794.5782	2.36	18:3/18:2	C45H80NO10
MGDG 36:4	2.22	796.5974	796.5939	4.39	18:2/18:2 and 18:3/18:1	C45H82NO10
MGDG 36:3	2.08	798.6124	798.6095	3.63	18:2/18:1	C45H84NO10
MGDG 36:2	2.08	800.6280	800.6251	3.60	18:1/18:1 and 18:0/18:2	C45H86NO10
MGDG 36:1	2.17	802.6376	802.6408	-4.02	18:0/18:1	C45H88NO10
**DGDG identified as [M+NH$_4$]$^+$**						
DGDG 34:3	2.30	932.6352	932.6310	4.50	18:3/16:0	C49H90NO15
DGDG 34:2	2.22	934.6483	934.6467	1.71	18:2/16:0 and 18:1-16:1	C49H92NO15
DGDG 34:1	2.30	936.6646	936.6623	2.46	18:1/16:0	C49H94NO15
DGDG 36:6	2.33	954.6177	954.6154	2.41	*	C51H88NO15
DGDG 36:5	2.30	956.6329	956.6310	1.93	18:3/18:2	C51H90NO15
DGDG 36:4	2.33	958.6511	958.6467	4.59	18:3/18:1 and 18:2/18:2	C51H92NO15
DGDG 36:3	2.30	960.6661	960.6623	3.96	18:2/18:1	C51H94NO15
DGDG 36:2	2.30	962.6816	962.6780	3.74	18:1/18:1	C51H96NO15
DGDG 36:1	2.33	964.6927	964.6936	-0.98	18:0/18:1	C51H98NO15
**Cer identified as [M+H]$^+$**						
Cer 34:3;O2	1.94	534.4906	534.4886	3.71	18:1;O2/16:2	C34H64NO3
Cer 40:1;O4	2.08	654.6059	654.6036	3.44	18:1;O3/22:0;O	C40H80NO5
Cer 40:0;O4	2.08	656.6202	656.6193	1.37	18:0;O3/22:0;O	C40H82NO5
Cer 41:1;O4	2.08	668.6223	668.6193	4.49	18:1;O3/23:0;O	C41H82NO5
Cer 41:0;O4	2.08	670.6357	670.6349	1.12	18:0;O3/23:0;O	C41H84NO5
Cer 42:1;O4	2.03	682.6375	682.6349	3.74	18:1;O3/24:0;O	C42H84NO5
Cer 43:1;O4	2.03	696.6531	696.6506	3.59	18:1;O3/25:0;O	C43H86NO5
**HexCer identified as [M+H]$^+$**						
HexCer 34:2;O2	2.25	698.5561	698.5571	-1.43	18:2;O2/16:0	C40H76NO8
HexCer 34:2;O3	2.28	714.5549	714.5520	4.06	18:2;O2/16:0;O	C40H76NO9
HexCer 38:1;O4	2.03	788.6272	788.6252	2.57	18:1;O3/20:0;O	C44H86NO10
HexCer 40:2;O3	2.19	798.6489	798.6459	3.76	18:2;O2/22:0;O	C46H88NO9
HexCer 40:1;O4	2.08	816.6597	816.6565	3.92	18:1;O3/22:0;O	C46H90NO10
HexCer 42:2;O3	2.08	826.6800	826.6772	3.38	18:2;O2/24:0;O	C48H92NO9
HexCer 42:1;O3	2.08	828.6915	828.6928	-1.57	18:1;O2/24:0;O	C48H94NO9
HexCer 42:1;O4	2.11	844.6905	844.6878	3.20	18:1;O3/24:0;O	C48H94NO10
HexCer 44:1;O4	2.08	872.7227	872.7191	4.15	18:1;O3/26:0;O	C50H98NO10
**ASG identified as [M+NH$_4$]$^+$**						
ASG 29:1;O;Glc;FA16:0	2.03	832.7067	832.7030	4.41	16:0	C51H94NO7
ASG 29:1;O;Glc;FA18:3	2.19	854.6882	854.6874	0.96	18:3	C53H92NO7
ASG 29:1;O;Glc;FA18:2	2.08	856.7063	856.7030	3.82	18:2	C53H94NO7
ASG 29:1;O;Glc;FA18:1	2.03	858.7217	858.7187	3.52	18:1	C53H96NO7
ASG 29:1;O;Glc;FA18:0	2.11	860.7303	860.7343	-4.68	18:0	C53H98NO7

All molecular species were identified by analyzing the respective MS/MS spectra, exact mass, and retention time, except the molecular species with an asterisk (*) identified only by exact mass and retention time. [a)] The fatty acyl chains of these species were identified

only in the positive-ion mode confirming the polar head group while no informative negative-ion mode MS/MS spectrum could be acquired. t_R, retention time. Lipid class abbreviation follows the LIPID MAPS updated nomenclature and shorthand notation, as C-atoms:number of double bond equivalents (DBE) and C-atoms:DBE;O-atoms for oxygenated lipids.[1] Likewise, molecular species with separator "/" mean that *sn*-position of acyl constituents is proven.[1] Abbreviations: PC, phosphatidylcholine; LPC, lysophosphatidylcholine; PE, phosphatidylethanolamine; LPE, lysophosphatidylethanolamine; PG, phosphatidylglycerol; MGDG, monoglycosyldiacylglycerol; DGDG, diglycosyldiacylglycerol; Cer, ceramide; HexCer, hexosylceramide; ASG, acylsterolglycoside.

Table S4. Total number of lipid species by polar lipid category and number of lipid species by polar lipid class identified in the olive seeds of the different sub-regions of Nelas, Portugal. Samples were collected in Nelas (Portugal) from six olive groves.

Sample group	VS_1	VS_2	VS_3	Sil	OB	VR
Phospholipids						
**PC**	**27**	**26**	**24**	**22**	**25**	**28**
**LPC**	**8**	**8**	**8**	**7**	**8**	**8**
**PE**	**12**	**12**	**10**	**12**	**11**	**12**
**LPE**	**4**	**4**	**4**	**4**	**3**	**4**
**PG**	**4**	**4**	**4**	**4**	**3**	**4**
No. lipid species	***55***	***54***	***50***	***49***	***50***	***56***
Glycolipids						
**MGDG**	**8**	**8**	**8**	**8**	**8**	**8**
**DGDG**	**9**	**9**	**8**	**8**	**9**	**9**
No. lipid species	***17***	***17***	***16***	***16***	***17***	***17***
Sphingolipids						
**Cer**	**6**	**6**	**7**	**5**	**6**	**6**
**HexCer**	**9**	**9**	**8**	**8**	**9**	**9**
No. lipid species	***15***	***15***	***15***	***13***	***15***	***15***
Sterol derivatives						
**ASG**	**5**	**4**	**5**	**5**	**5**	**4**
Total lipid species	***92***	***90***	***86***	***83***	***87***	***92***

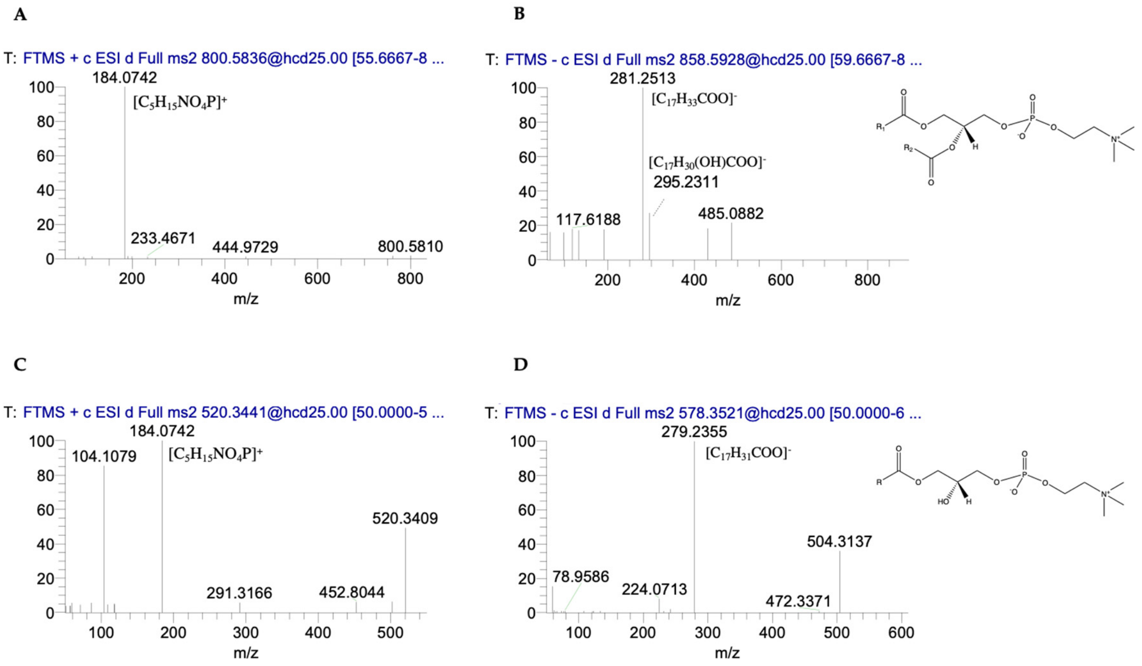

Figure S2. Illustrative LC-MS/MS spectra of the phosphatidylcholine and lysophosphatidylcholine classes identified in the olive seed cv. *Galega vulgar*. PC 36:3;O at *m/z* 800.58 as $[M + H]^+$ (A) and at *m/z* 858.59 as $[M + CH_3COO]^-$ (B). LPC 18:2 at *m/z* 520.34 as $[M + H]^+$ (C) and at *m/z* 578.35 as $[M + CH_3COO]^-$ (D). The notation C:DBE;O of the lipid species means the total number of carbon atoms (C), double bond equivalents (DBE), and the number of oxygen atoms (O). A general chemical structure of each class is also shown.

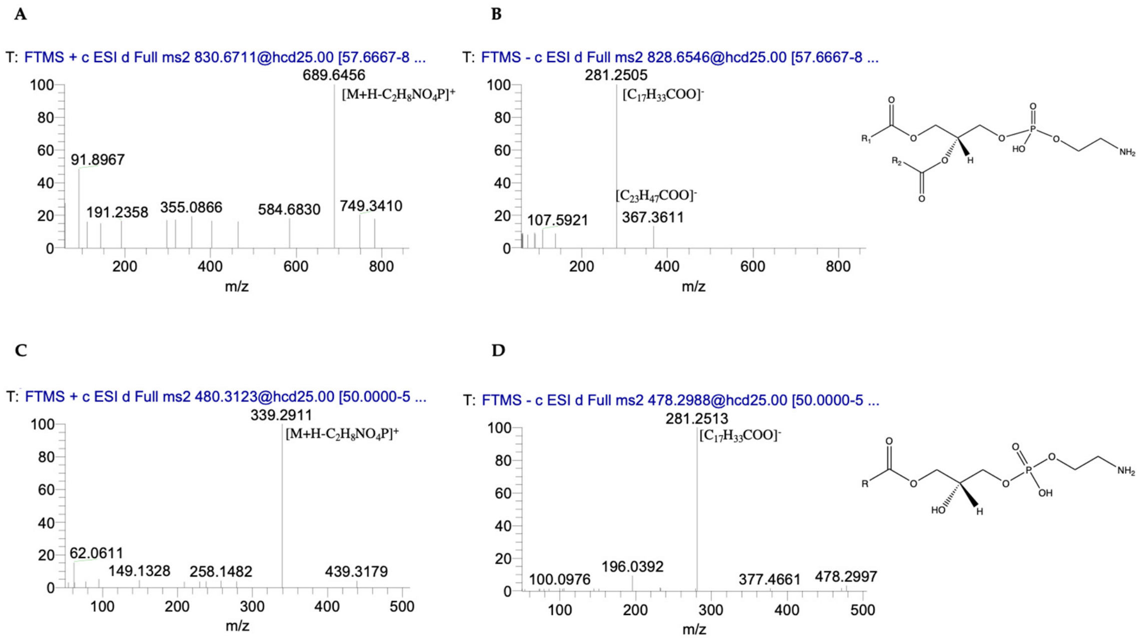

Figure S3. Illustrative LC-MS/MS spectra of the phosphatidylethanolamine and lysophosphatidylethanolamine classes identified in the olive seed cv. *Galega vulgar*. PE 42:1 at *m/z* 830.67 as $[M + H]^+$ (A) and at *m/z* 828.65 as $[M - H]^-$ (B). LPE 18:1 at *m/z* 480.31 as $[M + H]^+$ (C) and at *m/z* 478.30 as $[M - H]^-$ (D).

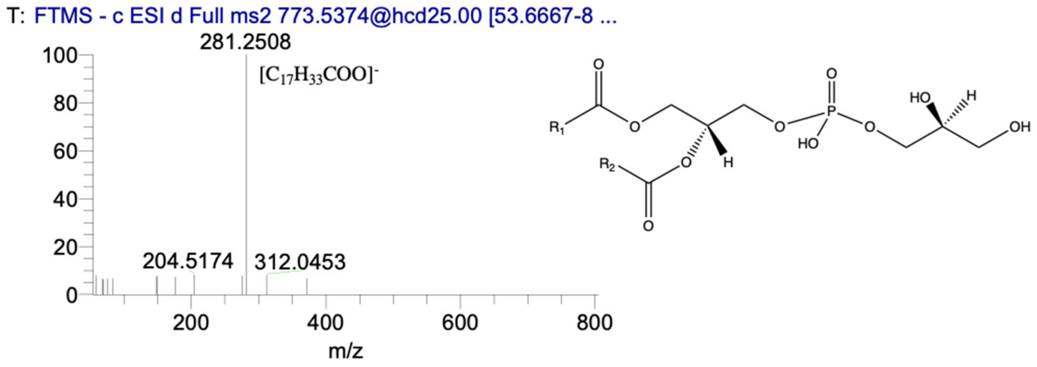

Figure S4. Illustrative LC-MS/MS spectrum of the phosphatidylglycerol class identified in the olive seed cv. *Galega vulgar*, PG 36:2 at *m/z* 773.53 as [M - H]$^-$.

A

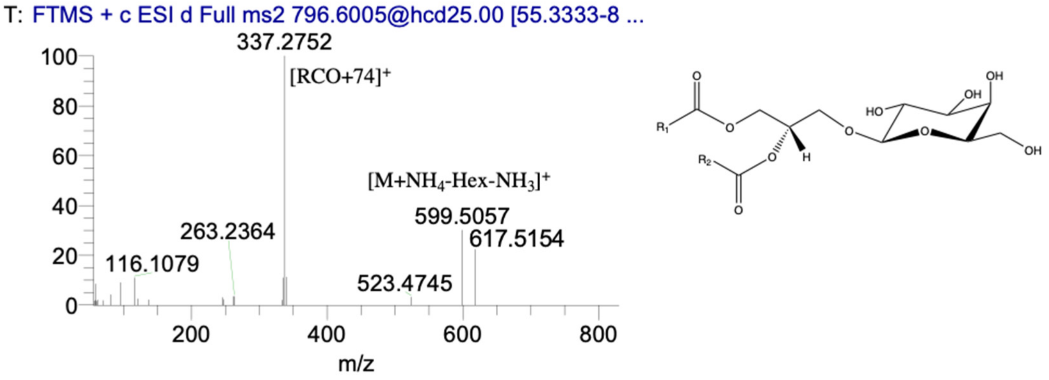

B

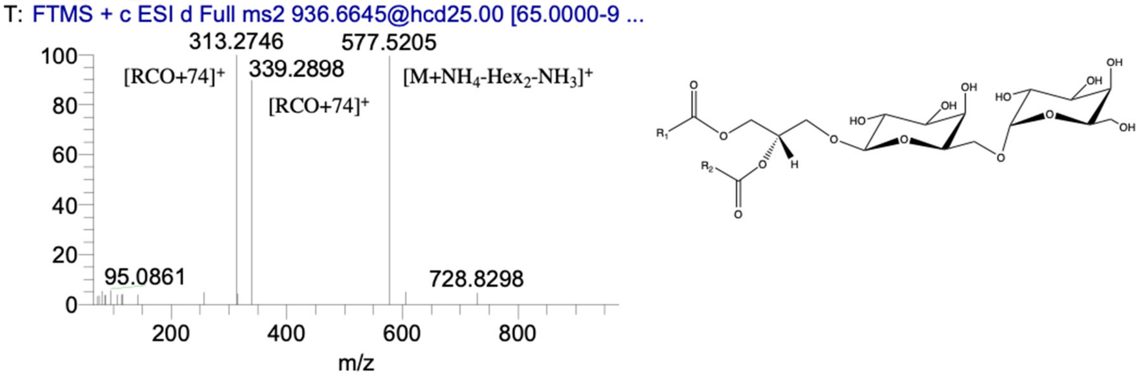

Figure S5. Illustrative LC-MS/MS spectra of the glyceroglycolipid classes identified in the olive seed cv. *Galega vulgar*: monoglycosyldiacylglycerol MGDG 36:4 at *m/z* 796.60 as $[M + NH_4]^+$ (A) and diglycosyldiacylglycerol DGDG 34:1 at *m/z* 936.66 as $[M + NH_4]^+$ (B).

A

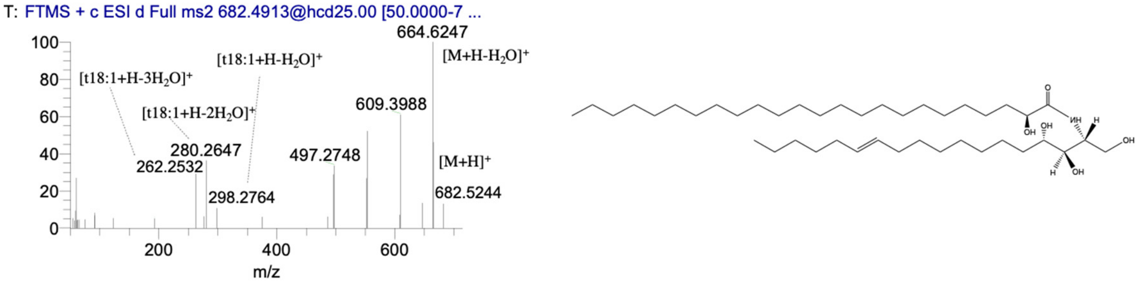

B

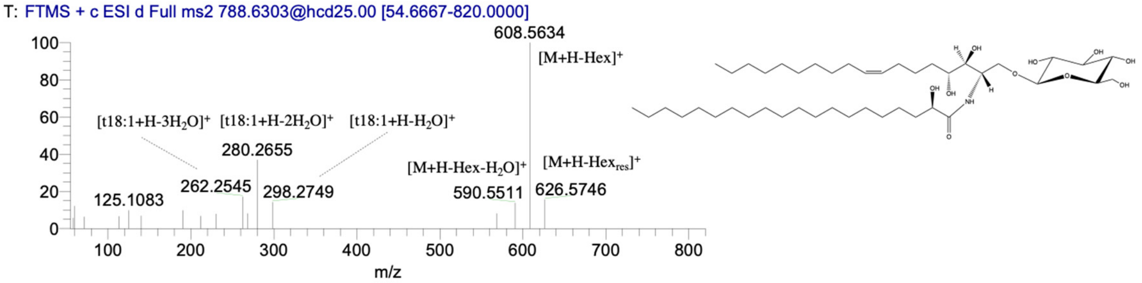

Figure S6. Illustrative LC-MS/MS spectra of the sphingolipid classes identified in the olive seed cv. *Galega vulgar*: Ceramide Cer 42:1;O4 at *m/z* 682.49 as $[M + H]^+$ (A) and hexosylceramide HexCer 38:1;O4 at *m/z* 788.63 as $[M + H]^+$ (B). The notation C:DBE;O of the lipid species means the total number of carbon atoms (C), double bond equivalents (DBE), and the number of oxygen atoms (O).

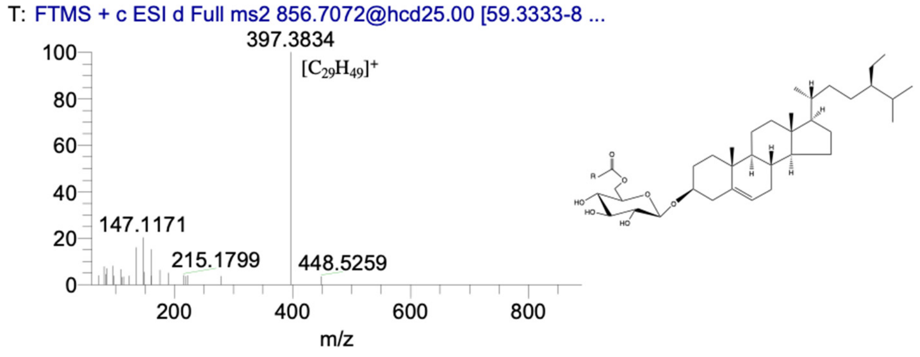

Figure S7. Illustrative LC-MS/MS spectrum of an acylsterolglycoside identified in the olive seed cv. *Galega vulgar*, 18:2-Glc-Sitosterol at *m/z* 856.71as [M + NH_4]$^+$.

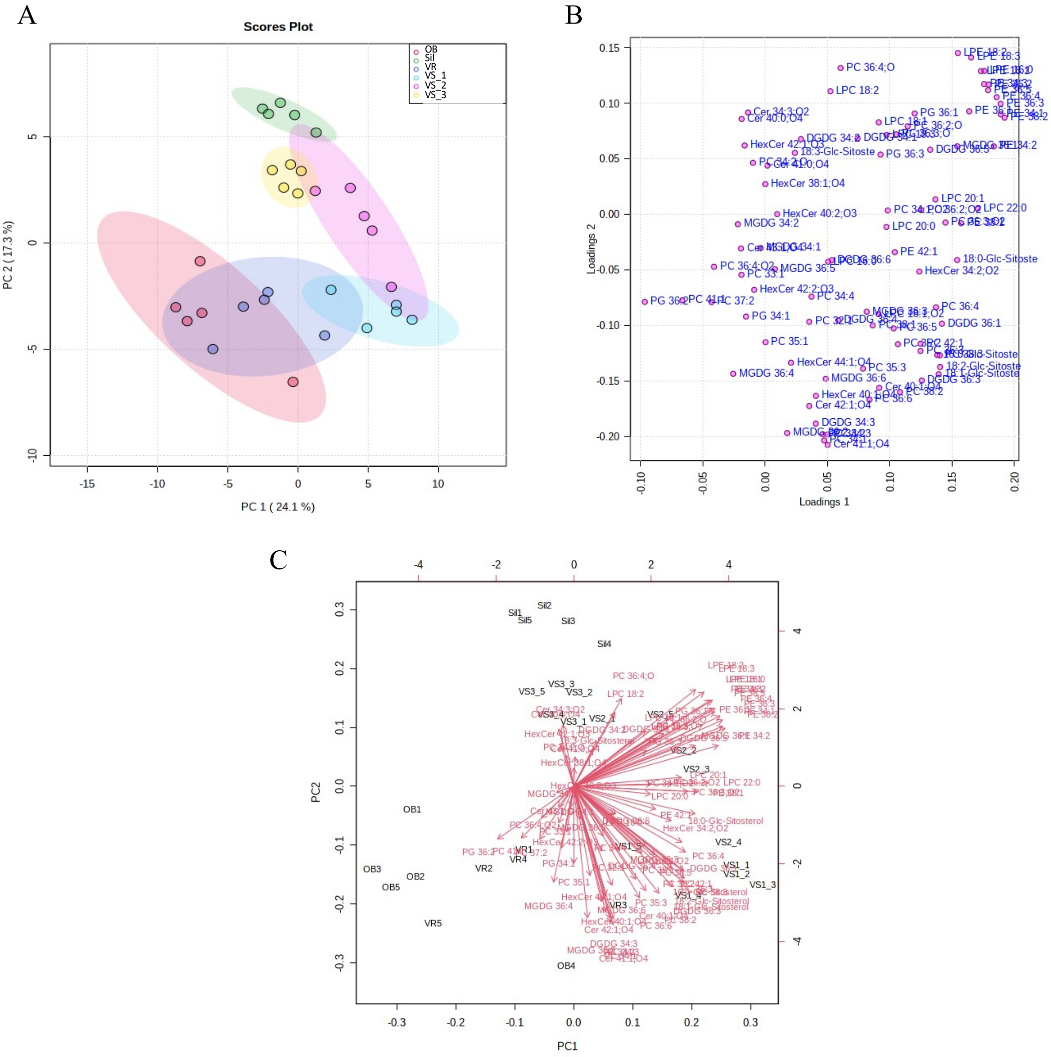

Figure S8. Principal components analysis (PCA) scores plot of the two first PC (PC2 versus PC1) (A), PCA loadings plot (B), and PCA biplot (C) performed on the whole standardized and log-transformed polar lipid species data set acquired by HILIC-LC-MS of olive seeds cv. *Galega vulgar* from different sub-regions of Nelas (Portugal): Vilar Seco_1 (VS_1), Vilar Seco_2 (VS_2), Vilar Seco_3 (VS_3), Silgueiros (Sil), Oliveira de Barreiros (OB), and Vila Ruiva (VR).

Table S5. Summary of ANOSIM analysis comparing the polar lipid profiles of olive seeds cv. *Galega vulgar* from different sub-regions of Nelas (Portugal): Vilar Seco_1 (VS_1), Vilar Seco_2 (VS_2), Vilar Seco_3 (VS_3), Silgueiros (Sil), Oliveira de Barreiros (OB), and Vila Ruiva (VR).

Groups	R Statistic	Significance Level
VS_1, VS_2	0.540	0.016
VS_1, VS_3	0.836	0.008
VS_1, Sil	1.00	0.008
VS_1, OB	1.00	0.008
VS_1, VR	0.844	0.008
VS_2, VS_3	0.720	0.008
VS_2, Sil	1.00	0.008
VS_2, OB	1.00	0.008
VS_2, VR	0.928	0.008
VS_3, Sil	1.00	0.008
VS_3, OB	1.00	0.008
VS_3, VR	0.820	0.008
Sil, OB	1.00	0.008
Sil, VR	1.00	0.008
OB, VR	0.996	0.008

Table S6. One-way analysis of variance (ANOVA) of the glog transformed and autoscaled HILIC-LC-MS data of polar lipid molecular species from olive seeds cv. *Galega vulgar*, followed by post-hoc Tukey's multiple comparison test and *p*-values correction for multiple testing using Benjamini–Hochberg false discovery rate (FDR, q values). Samples were collected in Nelas (Portugal) from six olive orchards located in Vilar Seco_1 (VS_1), Vilar Seco_2 (VS_2), Vilar Seco_3 (VS_3), Silgueiros (Sil), Oliveira de Barreiros (OB), and Vila Ruiva (VR).

**Lipid species**	**F value**	**FDR**	**Tukey's HSD**
Cer 34:3;O2	1496	3.68E-27	VS_3-OB; VS_3-Sil; VS_3-VR; VS_3-VS_1; VS_3-VS_2
PC 36:4	280,81	8.00E-19	VR-OB; VS_1-OB; VS_2-OB; VS_3-OB; VR-Sil; VS_1-Sil; VS_2-Sil; VS_3-Sil; VS_1-VR;
ASG 29:1;O;Glc;FA18:3	170,93	1.79E-16	Sil-OB; VR-OB; VS_1-OB; VS_2-OB; VR-Sil; VS_2-Sil; VS_1-VR; VS_3-VR; VS_2-VS_1;
PC 32:1	162,23	2.46E-16	VS_1-OB; VS_3-OB; VS_2-Sil; VS_3-Sil; VS_1-VR; VS_3-VR; VS_2-VS_1; VS_3-VS_1;
HexCer 40:1;O4	115,86	9.59E-15	Sil-OB; VS_1-OB; VS_3-OB; VR-Sil; VS_1-Sil; VS_2-Sil; VS_3-Sil; VS_3-VR; VS_2-
PC 34:1	106,25	2.15E-14	Sil-OB; VR-Sil; VS_1-Sil; VS_2-Sil; VS_3-Sil; VS_2-VS_1; VS_3-VS_1
PC 34:3	101,51	3.10E-14	Sil-OB; VR-Sil; VS_1-Sil; VS_2-Sil; VS_3-Sil; VS_2-VS_1; VS_3-VS_1
PE 38:1	95,529	5.42E-14	Sil-OB; VR-OB; VS_1-OB; VS_2-OB; VS_1-Sil; VS_2-Sil; VS_3-Sil; VS_1-VR; VS_2-VR;
Cer 41:1;O4	86,962	1.40E-13	Sil-OB; VR-Sil; VS_1-Sil; VS_2-Sil; VS_3-Sil; VS_3-VS_1
PC 34:2	81,477	2.62E-13	Sil-OB; VR-Sil; VS_1-Sil; VS_2-Sil; VS_3-Sil
PE 42:1	79,947	2.95E-13	Sil-OB; VR-OB; VS_1-OB; VS_2-OB; VS_3-OB; VS_3-Sil; VS_1-VR; VS_2-VR; VS_3-VR;
LPE 18:3	63,508	3.53E-12	Sil-OB; VR-OB; VS_1-OB; VS_2-OB; VS_3-OB; VR-Sil; VS_1-VR; VS_2-VR; VS_3-VR
PG 36:2	41,611	3.20E-10	Sil-OB; VR-OB; VS_1-OB; VS_2-OB; VS_3-OB
HexCer 44:1;O4	38,502	6.75E-10	Sil-OB; VR-Sil; VS_1-Sil; VS_2-Sil; VS_3-Sil; VS_3-VR
LPE 18:1	28,569	1.36E-08	Sil-OB; VR-OB; VS_1-OB; VS_2-OB; VS_3-OB; VR-Sil; VS_1-VR; VS_2-VR; VS_3-VR
LPE 16:0	28,114	1.50E-08	Sil-OB; VR-OB; VS_1-OB; VS_2-OB; VS_3-OB; VR-Sil; VS_1-VR; VS_2-VR; VS_3-VR
LPC 18:1;O2	27,422	1.82E-08	Sil-OB; VR-OB; VR-Sil; VS_1-Sil; VS_2-Sil; VS_3-Sil; VS_1-VR; VS_2-VR; VS_3-VR

PE 34:1	24,348	5.57E-08	Sil-OB; VR-OB; VS_1-OB; VS_2-OB; VS_3-OB; VR-Sil; VS_1-VR; VS_2-VR; VS_3-VR
PE 34:3	23,955	6.19E-08	Sil-OB; VS_1-OB; VS_2-OB; VS_3-OB; VR-Sil; VS_1-VR; VS_2-VR; VS_3-VR
PC 38:1	23,305	7.69E-08	VS_3-OB; VS_1-Sil; VS_3-Sil; VS_1-VR; VS_3-VR; VS_3-VS_1; VS_3-VS_2
PE 36:4	22,845	8.88E-08	Sil-OB; VR-OB; VS_1-OB; VS_2-OB; VS_3-OB; VR-Sil; VS_1-VR; VS_2-VR; VS_3-VR
PE 36:3	22,546	9.63E-08	Sil-OB; VR-OB; VS_1-OB; VS_2-OB; VS_3-OB; VR-Sil; VS_1-VR; VS_2-VR; VS_3-VR
PC 42:1	19,506	3.65E-07	VR-OB; VS_1-OB; VS_2-OB; VR-Sil; VS_1-Sil; VS_2-Sil; VS_3-VR; VS_3-VS_1; VS_3-
MGDG 36:4	18,731	5.11E-07	VS_2-OB; VR-Sil; VS_1-Sil; VS_2-Sil; VS_2-VR; VS_2-VS_1; VS_3-VS_1; VS_3-VS_2
LPE 18:2	18,595	5.24E-07	Sil-OB; VR-OB; VS_1-OB; VS_2-OB; VS_3-OB; VR-Sil; VS_2-VR; VS_3-VR

Table S7. Geographical and geological data of the studied sub-regions of Nelas (Portugal) and average climatological data of the Viseu Dão-Lafões regions where the locality of Nelas belongs.

Sub-region	GPS coordinates	Altitude (m)
Vilar Seco	40.57132, -7.85580	394
Silgueiros	40.563725, -7.95911	303
Oliveira de Barreiros	40.59634, -7.92430	392
Vila Ruiva	40.540990, -7.76605	448
**Viseu Dão-Lafões region, year 2016**		
Temperature	12.3 ºC	
Drought index (SPI)	1.4	
Relative humidity	77 %	
Accumulated precipitation	1410.9 mm	
Daily thermal range	9.4 ºC	
Global radiation	152 $W.m^{-2}$	

SPI, standardized precipitation index. Source for GPS coordinates and altitude: https://geoportal.lneg.pt/mapa/. Source for climatological data: http://portaldoclima.pt/

Reference

(1) Liebisch, G.; Fahy, E.; Aoki, J.; Dennis, E. A.; Durand, T.; Ejsing, C. S.; Fedorova, M.; Feussner, I.; Griffiths, W. J.; Köfeler, H.; Merrill, A. H.; Murphy, R. C.; O'Donnell, V. B.; Oskolkova, O.; Subramaniam, S.; Wakelam, M. J. O.; Spener, F. Update on LIPID MAPS Classification, Nomenclature, and Shorthand Notation for MS-Derived Lipid Structures. *Journal of Lipid Research* **2020**, *61* (12), 1539–1555. https://doi.org/10.1194/jlr.S120001025.
